# Group Mindfulness-Integrated Cognitive Behavior Therapy (MiCBT) Reduces Depression and Anxiety and Improves Flourishing in a Transdiagnostic Primary Care Sample Compared to Treatment-as-Usual: A Randomized Controlled Trial

**DOI:** 10.3389/fpsyt.2022.815170

**Published:** 2022-05-31

**Authors:** Sarah E. B. Francis, Frances Shawyer, Bruno Cayoun, Joanne Enticott, Graham N. Meadows

**Affiliations:** ^1^Southern Synergy, Department of Psychiatry, School of Clinical Sciences at Monash Health, Monash University, Melbourne, VIC, Australia; ^2^Mindfulness-Integrated Cognitive Behavior Therapy Institute, Hobart, TAS, Australia; ^3^Monash Centre for Health Research and Implementation, School of Public Health and Preventive Medicine, Monash University, Melbourne, VIC, Australia; ^4^Mental Health Program, Monash Health, Melbourne, VIC, Australia; ^5^Melbourne School of Population and Global Health, University of Melbourne, Parkville, VIC, Australia

**Keywords:** mindfulness, equanimity, transdiagnostic treatment, interoception, Mindfulness-integrated Cognitive Behavior Therapy (MiCBT)

## Abstract

**Objectives:**

This study investigated the effectiveness of a group-based 8-week intervention, Mindfulness-integrated Cognitive Behavior Therapy (MiCBT), to decrease psychological distress and increase wellbeing in a heterogeneous population in primary health care. MiCBT focuses on the importance of interoception and its interaction with cognition in emotional experience. These interactions are represented in the co-emergence model of reinforcement, in which non-reactivity (equanimity) to interoceptive signals facilitates adaptive behavior.

**Methods:**

Participants (*n* = 125, aged 20–72) were randomized to two groups (MiCBT), and treatment-as-usual (TAU). Outcomes were assessed at pre-, mid-, and post-intervention and at 6-month follow-up. The primary outcome was psychological distress, measured by the Depression, Anxiety and Stress Scale (DASS-21). Secondary outcome measures were the Kessler Psychological Distress Scale-10 (K10), Satisfaction with Life Scale (SWLS), and Flourishing Scale (FS). Mediator or process measures of interoceptive awareness, metacognitive awareness (decentering), equanimity, and social functioning were included to investigate putative mediators.

**Results:**

The MiCBT intervention significantly reduced DASS-21 scores at mid and post-treatment and the gains were maintained at 6-month follow-up (*p* < 0.0001, *d* = 0.38). Flourishing scores also showed significant improvement post-treatment and at 6-month follow-up (*d* = 0.24, *p* < 0.0001). All measures selected showed a similar pattern of positive change, with the exception of the SWLS, which failed to reach significance. Mediation analysis suggested equanimity to be the most influential mediator of the primary outcome.

**Conclusions:**

The results support the effectiveness of MiCBT in creating rapid and sustainable reduction of psychological distress and improvement in flourishing in a primary mental health care setting with heterogenous groups. These promising results support the scaled-up implementation of this intervention.

**Clinical Trial Registration:**

This trial is registered with the Australian and New Zealand Clinical Trial Registry: https://www.anzctr.org.au/ACTRN12617000061336.

## Introduction

Comorbidities are common in mental health conditions (1–3) and have been associated with greater use of health services but also worse outcomes (4). In order to address co-morbidities, transdiagnostic protocols—protocols that can be applied across a range of processes such as cognitive-behavioral, interpersonal, or biological—have been attracting attention (5, 6). A number of psychological processes are proposed to be transdiagnostic including ruminative negative thinking, biased reasoning, selective attention to stimuli (both internal and external) and avoidance (5, 7). Interventions that can address such psychological processes could therefore be used to treat multiple conditions, both within and across individuals, obviating the need to tailor the content to particular diagnostic groups (8, 9). Despite recent attention to transdiagnostic approaches, therapies for mental health conditions, including traditional cognitive-behavioral therapies (CBT), and more recently mindfulness-based therapies are, in the main, disorder specific following a medical diagnostic model (6).

Mindfulness-integrated Cognitive Behavior Therapy [MiCBT—(10–12)] potentially makes a valuable contribution to the emerging suite of transdiagnostic interventions because it addresses a number of transdiagnostic processes including metacognitive awareness (discernment and regulation of reasoning), selective attention to stimuli (both internal and external), and avoidance. Additionally, MiCBT addresses emotion regulation which has been proposed to be a transdiagnostic factor (13). Emotion regulation is addressed through the development of interoceptive awareness (awareness of body sensations) together with equanimity. Equanimity has been defined “as an even-minded mental state or dispositional tendency toward all experiences or objects, regardless of their origin or their affective valence (pleasant, unpleasant, or neutral)” [(14), p. 4] and could be simplistically defined as non-reactivity. MiCBT proposes that emotional reactivity is based on conditioning to interoceptive signals and that the development of equanimity to interoceptive signals through exposure (e.g., while scanning the body during mindfulness meditation) facilitates extinction processes.

Both interoceptive signals and the cognitive evaluation of the internal and external environment are recognized as contributing to an emotional experience (15–17). In the case of a fear experience that is evaluated as threatening, the level of arousal increases the production of interoceptive signals as part of the emotional experience thus setting up expectations for future similar situations. If the interoceptive signals can be cognitively re-evaluated as non-threatening then the learned response can be extinguished (18). It is now established that interoceptive awareness is impaired in people with mental health disorders (19, 20)—for example, with heightened interoception (e.g., panic disorder) or reduced interoception (e.g., dissociative disorder). It has also been suggested that atypical interoception may be associated with vulnerability to psychopathology (17).

In MiCBT, interoceptive awareness and equanimity are achieved through a process of systematic exposure to body sensations using a sequence of body scanning exercises to practice and cultivate equanimity toward body sensations. Since habitual reactivity is a common factor shared by numerous mental health conditions (21), treatments which address these underlying processes are likely to have transdiagnostic effects. As MiCBT places great emphasis on increasing interoceptive awareness and desensitization, it is expected to improve emotion regulation across a range of emotional disorders, including those with complex comorbidities. MiCBT is designed for acute and chronic mental health conditions.

**Figure 1:**
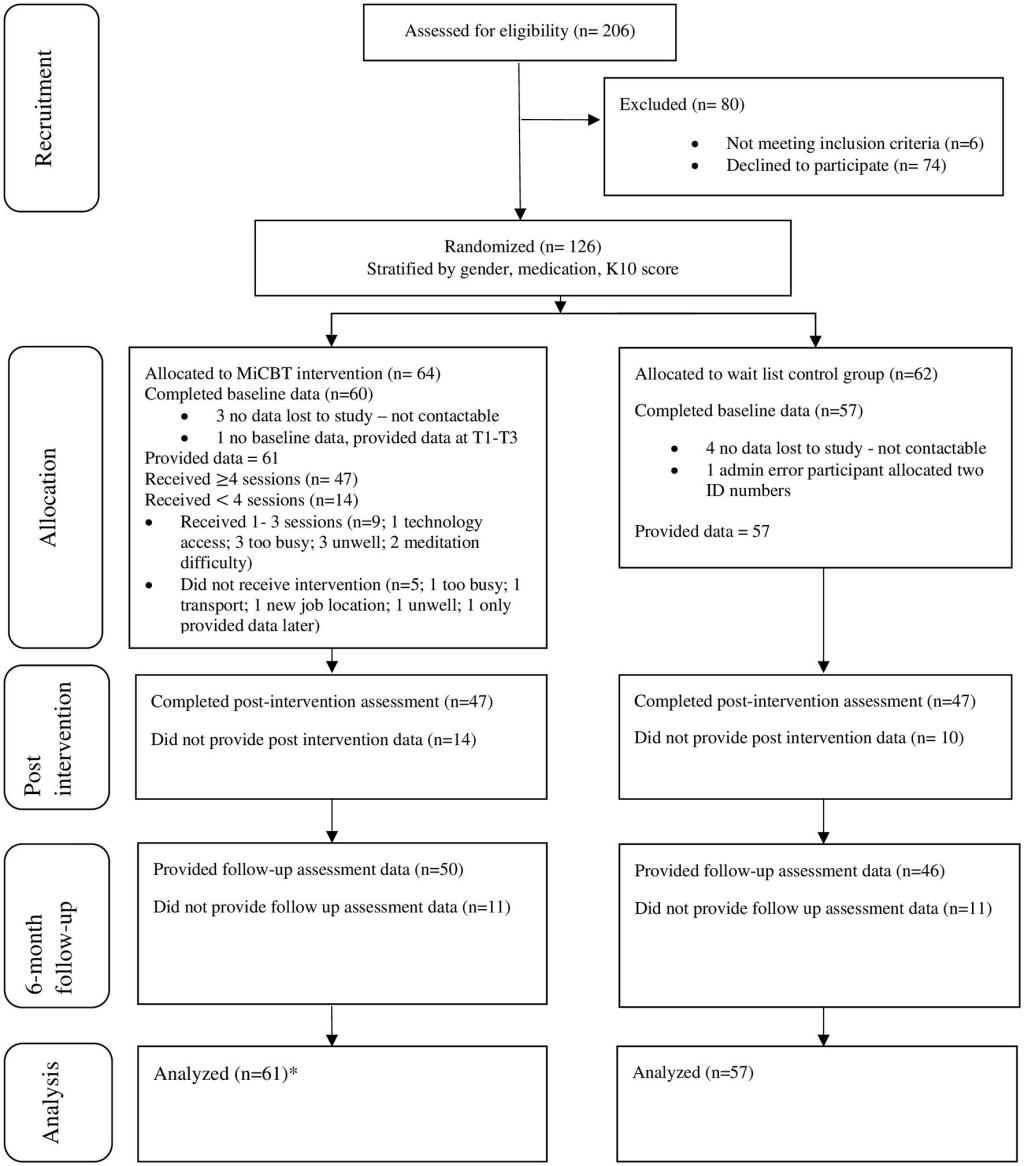
CONSORT flow diagram. ^*^One participant provided data at post and follow up only.

The rationale for MiCBT, described in detail in the protocol paper for the current study (22, 23) and elsewhere (10–12) is based on the co-emergence model of reinforcement (10, 24) which is explicitly taught during the intervention [see (22, 23) Figure 1, p. 3]. The model proposes that following a sensory stimulus (either from external sources through sight, smell, touch, taste, or hearing or from internal sources through thinking, remembering or interoception) an evaluative process takes place. Sensory events that are deemed to be personally relevant result in spontaneous co-emerging interoception, which directs behavior. According to the co-emergence model, behavior is moderated by the quality and strength of co-emerging interoceptive experience (body sensations), where unpleasant body sensations are more likely to be the antecedents of avoidant behavior, while pleasant sensations are more likely to generate seeking behaviors. This is consistent with Barrett's theory of emotion which proposes that the tendency to react is proportional to the strength of affect (25), and thus emotions are not considered to be reactions to the world but dynamic constructions of it (26).

MiCBT integrates meditation skills adopted from the Vipassana (insight) tradition taught by U Ba Khin and Satya Narayan Goenka (10). Accordingly, MiCBT provides a structured sequence of practices: (1) body awareness through progressive muscle relaxation and attention to posture and movement in daily activity, followed by (2) mindfulness of breath to increase attention regulation, metacognitive awareness and response inhibition, (3) body-scanning methods to develop deep levels of interoceptive awareness and equanimity (body-scanning methods comprise 75% of the meditations in MiCBT), and (4) loving kindness meditation to increase empathy and compassion for self and others.

MiCBT Involves four stages. Stage 1 mostly consists of learning the above meditation skills to self-regulate. In stages 2 and 3, the exposure methods implemented include graded imaginal and *in vivo* exposure to increase tolerance of interoceptive signals while reducing reactivity to avoided situations and conflictual interpersonal situations. In the fourth stage, to assist in preventing relapse, exposure is applied to potential harmful behaviors which patients learn to prevent out of compassion for themselves and others. According to the MiCBT rationale, reducing reactivity assists with improved general functioning, including social functioning and connectedness with others, thus enhancing wellbeing and flourishing (27, 28), which in turn helps prevent relapse (28).

Evidence for the efficacy of MiCBT is building across different conditions, including depression, anxiety and depression in individuals with multiple sclerosis (29, 30), anxiety and depression during pregnancy (31), sports anxiety (32), pain and pain self-efficacy in patients with breast cancer (33), and chronic pain in the general population (34). In a very recent study, post-partum depression was demonstrated to be ameliorated in a group who received MiCBT and changes were detected at a chromosomal level (35). An uncontrolled unpublished pilot study of group MiCBT for patients with mixed diagnoses conducted in a private psychology practice setting (*n* = 69) demonstrated significant reductions in psychological distress across disorders (36). However, to our knowledge, the application of MiCBT as a group intervention in a transdiagnostic psychiatric population has not been subject to controlled investigation.

The aim of the current study was to investigate the effectiveness of MiCBT as a transdiagnostic group-based therapy to decrease psychological distress for patients with a range of mild to severe mental health disorders in a primary health care setting in real world conditions. The hypotheses were that, compared to the control group, the MiCBT group would show a greater decrease in self-report assessments of depression, anxiety and stress, and greater improvement in life satisfaction and flourishing. It was also hypothesized that the improvements would be maintained over a 6-month follow-up period. Additionally, it was hypothesized that improvements in the clinical measures as a function of MiCBT relative to the control group would be mediated by (a) improvements in metacognitive and interoceptive awareness, and (b) improvement in equanimity. We also hypothesized that changes in life satisfaction and flourishing scores would be mediated more by improvements in interpersonal skills compared to awareness and equanimity.

## Methods

Participants were randomized to either the MiCBT or the treatment-as-usual (TAU) control condition. Participants on both conditions continued with treatment-as-usual including medications and/or psychological therapy. The MiCBT intervention was offered to control group participants following the completion of the 6-month follow-up assessment. This study was granted ethical approval by the Monash University Human Research Ethics Committee (Project Number: CF16/2278-−2016001131). All participants provided written informed consent prior to their inclusion in the study.

### Recruitment

Information about the study was circulated to local medical and psychology clinics and rolling recruitment took place over 2 years through referrals from local general medical practitioners, psychiatrists, and psychologists. Inclusion criteria were: age 18–75; fluent in English; and with a Kessler Psychological Distress Scale-10 (K10) score of 20 or more. Exclusion criteria included: <18 years; non-English speakers; current psychotic symptoms, current drug or alcohol dependency, current diagnosis of borderline or antisocial personality disorder, pervasive developmental delay, organic mental disorder, or prescribed more than 20 mg diazepam equivalent per day. Patients who were referred to the study were interviewed to confirm eligibility and to gain written consent. A total of 206 patients expressed interest in the study and were assessed. Of the 80 participants excluded from the study, 6 did not meet criteria and 74 declined to participate (see Figure 1).

### Sample Size

The trial is powered to detect significant differences relevant to the primary outcome. A power analysis (G-power 3.1) for medium effect (Cohen's *d* = 0.5) was conducted with power of 0.8, and using the conventional significance level (0.05), resulting in a desired sample of 102 (51 participants in each group). Consistent with previous studies [e.g., (37)] allowing for a conservatively estimated attrition rate of 20% to follow up, we aimed to recruit a sample of 120. Based on the projected sample size of 102, power of 0.8, and zero, small, medium or large effect sizes conditions for the indirect effect, the mediation analyses are well-powered to detect medium beta paths between the independent variable (IV) and the mediator and between the mediator and the dependent variable (DV) (β = 0.39) using bootstrapping and the Sobel test (38). Data were collected using online self-report measures with the Qualtrics platform at the four measurement points: baseline, mid-intervention, post-intervention, and 6-months follow-up.

### Randomization

Participants were assigned a unique identification number and allocated to either the control or the MiCBT group. Blocked randomization was conducted using Stata (version 14SE) by means of alternating treatment group allocation based on blocks to yield treatment groups with balanced proportions of participants from respective blocks. A sequence of treatments (treatment or control) was created randomly permuted in blocks of size 4. Blocks of size 4 were chosen to ensure a balance of treatments in all group sessions, as groups of size 8 could begin as soon as 16 participants were recruited.

Randomization was also stratified based on three stratification variables, each having two values: K10 score (20–29, mild to moderate; ≥ 30, severe); psychotropic medication (yes/no) and gender (female/male). Therefore, a randomization schedule was generated for each of the 8 strata (2 × 2 × 2):


$$\begin{array}{l} \begin{array}{ccc} \text{F } & \text{N } & 20+\\ \text{F } & \text{N } & 30+\\ \text{F } & \text{Y } & 20+\\ \text{F } & \text{Y } & 30+\\ \text{M } & \text{N } & 20+\\ \text{M } & \text{N } & 30+\\ \text{M } & \text{Y } & 20+\\ \text{M } & \text{Y } & 30+\\ \; \end{array} \end{array}$$

Stata Version 14 SE generated the randomized schedules for this study with 2 arms and with a sample size of 400, a block size of 4, or 100 blocks of 4. Randomization was conducted by a researcher who was independent from the recruitment, assessment and treatment of participants.

### Participants

In total, 125 participants provided signed informed consent and were randomized to a treatment group (one participant was mistakenly allocated two identification numbers). However, 7 participants were lost to the study before contributing any data leaving 118 participants. Of these, 86 identified as female (72.9%), 31 as male (26.3%), and one as “other” (0.8%); the age range was 20–72 years. As per inclusion criteria, all participants had a K10 score of ≥ 20 on referral and this was administered again at the start of the study. Demographic and baseline clinical characteristics are shown in Table 1.

**Table 1:** Participant characteristics.

**Variable**	**MiCBT group**	**Control group**
**Gender:** ***n*** **(%)**		
Male	15 (24.6)	16 (28.1)
Female	46 (75.4)	40 (70.2)
Other		1 (1.8)
**Age:** ***n*** **(%)**		
18–34	21 (34.4)	18 (31.6)
35–49	15 (24.6)	24 (42.1)
50–75	25 (41.0)	15 (26.3)
**Nationality:** ***n*** **(%)**		
Born in Australia	49 (80.3)	47 (82.5)
Born outside Australia	12 (19.7)	10 (17.5)
**Marital status:** ***n*** **(%)**		
Married/in a relationship	25 (55.7)	32 (56.1)
Single/divorced/separated	34 (41)	23 (40.4)
Other	2 (3.3)	2 (3.5)
**Education:** ***n*** **(%)**		
High school education only	12 (19.7)	4 (7)
Post high school education	49 (80.3)	53 (93)
Taking Psychotropic medications *n* (%)	26 (70)	35 (66.7)
Pre-trial meditation practice *n* (%)	18 (30)	19 (33.3)
K10 score at referral: mean (SD)	29.4 (6.1)	29.3 (5.6)
K10 score T0	27.7 (7.0)	29.0 (6.3)
**K10 score at referral** ***n*** **(%)**		
<30 (mild-moderate)	30 (49.2)	30 (52.6)
≥30 (severe)	31 (50.8)	27 (47.4)
**K10 score T0** ***n*** **(%)**		
<30 (mild-moderate)	38 (62.3)	28 (49)
≥30 (severe)	23 (37.7)	29 (51)

Participants were typically well educated, 87% having tertiary education. In both groups ~70% were taking some form of psychotropic medication; 30% had some meditation experience and/or currently had some meditation practice.

### Participant Timeline

Referring General Medical Practitioners administered an initial K10 as part of the referral process. Participants were interviewed for eligibility and those who were eligible were randomized to either the control or MiCBT condition. Baseline assessments were administered post-randomization, 1 week before the commencement of the program so that the assessment timeframe could be standardized across participants and to address the significant variation in the period between recruitment and the start of the intervention. This was expected to achieve maximum sensitivity to change taking account of fluctuating mental health conditions. The flow of participants through the study is outlined in Figure 1.

### Measures

All outcome and process measures listed below are psychometrically acceptable self-report questionnaires and are described more fully in the protocol paper (22, 23). Both the K10 and the DASS-21 were used in the current study to measure psychological distress. The DASS-21 however provides both a global measure (total score) and measures of depression, anxiety and stress as separate subscales. The DASS-21 scores were the primary focus of the analysis.

#### Primary Outcome Measure

##### Depression, Anxiety, and Stress Scale (DASS-21)

The DASS-21 is a 21-item self-report questionnaire designed to measure three domains: depression, anxiety and stress experienced in the previous week (39). Each subscale contains 7 items, and items are rated on a four-point Likert scale. The DASS-21 is reported to have high internal consistency for each of the subscales (Cronbach's alpha: depression, α = 0.88; anxiety, α = 0.82; stress, α = 0.90; total scale α = 0.93), alongside evidence of good convergent and discriminant validity (40). Factor analysis supports the use of the total score as a measure of general psychological distress (40). The DASS-21 total score was used as the primary outcome while also reporting findings for the three subscales. In accordance with the manual (41) the scores on the DASS-21 were doubled to convert them to the full scale DASS scores and enable interpretation using the norms for the DASS-42. The manual provides recommended cutoffs for conventional severity labels derived from z score conversions from the normative sample (normal, mild, moderate, severe, or extreme range) (41). Higher scores indicate more severe symptoms relative to the population.

#### Secondary Outcome Measures

##### Kessler Psychological Distress Scale (K10)

The K10 is a 10-question screening scale of psychological distress that focuses on emotional problems experienced in the last 4 weeks and is shown to discriminate severity of psychological distress between clinically significant disorders as defined in the DSM-IV and non-clinically significant disorders. Several Australian studies using national survey data support the psychometric properties of the K10 including its validity as a measure of distress and as screen for mental health disorders, particularly anxiety and depressive conditions (1, 42, 43). This measure was chosen because it is commonly used by General Practitioners in Australia as a screening tool to identify those eligible for subsidized psychological treatment.

##### Satisfaction With Life Scale (SWLS)

This is a 5-item self-report measure of global judgment of life satisfaction (41). The SWLS uses a 7-point Likert scale from 1 (strongly disagree) to 7 (strongly agree). The range of possible scores is from minimal satisfaction with life (5) to very high satisfaction with life. Higher scores indicate higher levels of life satisfaction. The SWLS has been shown to have convergent validity with other self-report measures of life satisfaction, including the Philadelphia Geriatric Center Morale Scale. The SWLS appears to tap a single life satisfaction factor. Internal consistency is good with Cronbach's alpha of α = 0.87.

##### The Flourishing Scale (FS)

The FS is an 8-item self-report measure of psychological wellbeing, designed to tap self-perception of aspects of wellbeing such as optimism, positive relationships and self-esteem (44). It uses a 7-point Likert scale with responses from strong disagreement to strong agreement. Scores range from 8 to 56 and high scores indicate positive self-appraisal of psychological wellbeing. The scale developers report strong correlations with other psychological wellbeing scales and good internal consistency with Cronbach's alpha of α = 0.87.

#### Process Measures

Process measures were chosen to best reflect the aspects of mindfulness which are the focus of MiCBT.

##### Multi-Dimensional Assessment of Interoceptive Awareness (MAIA)

The MAIA is a 32-item self-report measure of interoceptive awareness (45). The MAIA has eight subscales, all reflecting different aspects of interoception; Noticing, Not-Distracting, Not-Worrying, Attention Regulation, Emotional Awareness, Self-Regulation, Body Awareness, Trusting (45). We therefore decided to use total MAIA scores in the current study. Higher scores indicate higher levels of interoceptive awareness. The internal-consistency reliability of each of the eight subscales was reported by the scale developers as ranging from α = 0.66 to 0.87.

##### The Non-attachment Scale (NAS)

The NAS is a 30-item self-report scale. It is a unidimensional measure of attachment as the term is used in Buddhism tapping the construct of being equanimous, flexible and receptive (46). It uses a 6-point Likert scale ranging from 1 (disagree strongly) to 6 (agree strongly). Higher scores indicate higher levels of non-attachment. The NAS is shown to correlate with a quality of consciousness related to constructs such as emotion regulation, interpersonal effectiveness, wellbeing and mental health. Internal consistency levels are > α = 0.80 (Cronbach's alpha).

##### The Experiences Questionnaire (EQ)

This is a 20-item self-report measure of decentering; the ability to observe thoughts and feelings with objectivity rather than identifying with experiences (47). Higher scores indicate higher levels of decentering. Metacognitive awareness is a key element of decentering, conceptualized as a protective factor assisting with resilience to depressive relapse (48–50). For the purposes of this paper, metacognitive awareness and decentering are used interchangeably. The EQ is a relatively new measure whose psychometric properties are still being investigated. The initial psychometric evaluation of the EQ supported its concurrent and divergent validity with significant positive correlations reported with a measure of cognitive reappraisal and significant negative correlations with brooding rumination, experiential avoidance, emotional suppression, and depressive and anxiety symptoms (47). In a later Spanish study, the EQ scale was found to have good internal consistency (Cronbach's alpha α = 0.89) and ability to differentiate between psychiatric and non-psychiatric patients. Convergent validity was evidenced by significant correlations with the Mindfulness Attention and Awareness Scale (MAAS) (*r* > 0.58), and the Five Factor Mindfulness Questionnaire (FFMQ) (*r* > 0.46) while divergent validity was evidenced by significant negative correlations with measures of anxiety, depression, stress and experiential avoidance (*r* < −0.35) (51).

##### Mindfulness-Based Self-Efficacy Scale-Revised (MSES-R)

The MSES is a 22-item self-report measure designed to assess the changes in levels of perceived self-efficacy due to mindfulness-based therapy (52). Higher scores indicate higher levels of mindfulness-based self-efficacy. The six subscales were separated into two clusters for the purposes of the current study; Emotion Regulation, Distress Tolerance, Equanimity (considered here as measuring aspects of equanimity; MSES-R-E), and Taking Responsibility, Social Skills, and Interpersonal effectiveness (considered here as measuring aspects of interpersonal skills; MSES-R-S). The MSES-R total shows high internal consistency (Cronbach's alpha α = 0.86); has a good divergent validity, inverse relationships with the DASS-21, and good concurrent validity with the Freiberg Mindfulness Inventory (FMI), the MAAS, the Kentucky Inventory of Mindfulness (KIMS), and the FFMQ. A recent study (52) reported high reliability using McDonald's ω (ω = 0.87).

#### Other Measures

##### Meditation Practice

Participants recorded daily meditation practice by recording the number of minutes per practice session and the number of practice sessions per week on a prescribed form in their program notes. The weekly total was collected by the researcher at each session on an individual form not visible to other participants.

##### Adverse Effects

Each week participants were asked about their practice so that any issues raised could be addressed. In a few instances minor modifications to practice were recommended such as continuing with an exercise a little longer before moving to the next exercise. At 6-month follow-up, questions were included regarding any adverse experiences believed to be due to meditating. We took these questions from a recent study of experiences that occur in association with meditation practices (53). The questions were:

1. “Did you have any unexpected, challenging, or difficult experiences that you associate with your practice of meditation?”
2. “How did these experiences impact your life”?

### Procedure

All participants completed the outcome and process measures 1 week before the start of the MiCBT group intervention (T0), after week 4 (T1), at week 8 (T2, post-intervention), and then again after a 6-month follow up period (T3). Demographic questions were included at T0, program evaluation questions at T2, and at T3. There were questions about meditation practice in the last 6 months as well as about any adverse effects. Measures were administered online using Qualtrics survey software, except for the amount of meditation practice, which was recorded by participants as described above. Participants were emailed a link to the online measures utilizing their unique identification number. All data was collected online (except meditation practice hours).

### Intervention Groups

The intervention protocol (22, 23) was adapted from the published protocols (10, 11) and consisted of 8 weekly 2 h classes. Adaptations from the original 8-week protocol included: (a) using the most recent publicly available audio instructions from Cayoun (11) (used with permission) and (b) individual sessions between groups were not routinely offered. In accordance with the protocol, a guided meditation was conducted at each session to introduce the practice required for the following week. The remainder of the time in the sessions was used for a review and discussion about practice experiences and psychoeducation, including the rationale for practices and homework tasks. Recommended weekly homework tasks included two half-hour meditation sessions per day (7 h per week). Intervention group sizes varied between 8 and 13 participants. The intervention was delivered by a registered psychologist trained in MiCBT who had been implementing MiCBT regularly over the past 10 years. Handouts for each session were provided as a workbook at the start of treatment. The handouts included practice record sheets, worksheets, and a list of homework tasks. The treatment outline is depicted in Table 2.

**Table 2:** Four stages of the MiCBT 8-week program.

**1. Personal stage (weeks 1, 2 and 3)**	**2. Exposure stage (weeks 4 and 5)**	**3. Interpersonal stage (week 6)**	**4. Empathic stage (weeks 7 and 8)**
• Progressive muscle relaxation and attention to body posture • Mindfulness of breath (attending to breath) • Body scanning (Burmese Vipassana tradition) • Co-emergence model of reinforcement	• Advanced body scanning • Overcoming avoidance • Integrating mindfulness and CBT—exposure tasks to address avoidance	• Advanced body scanning • Moving from focus on self to others; exposure to interpersonal difficulties • Mindful assertiveness • Taking responsibility for own experience in interpersonal situations; exposure tasks for interpersonal avoidance	• More advanced and flowing body scanning • Empathy and compassion with loving kindness meditation • Non-attachment to sense of self • Ethics—minimizing harm to self and others

Control group participants received treatment as usual during the 8-week intervention period and until the end of the 6-month follow up at which time the MiCBT intervention was offered (32 participants took up the offer). Treatment as usual for mental health conditions in Australian primary care is largely delivered through medical General Practitioners including assessment, treatment planning, direct treatment (such as medication and initial focused psychological strategies) and referral to specialist private services including psychiatry, psychology and other allied health services. Australian Medicare, designed as a universal health insurance scheme, provides Australian residents with rebates to help cover the cost of medical services from private health providers, including General Practitioners and specialist providers, based on scheduled items of care (the Medicare Benefits Schedule). Co-payments from patients are unrestricted and practitioners are free to choose their practice location. Standard treatment in primary care is regulated by National Standards (54) adherence to which is upheld by regular accreditation.

### Statistical Analyses

#### Main Analyses

We used mixed-model repeated measures to account for the correlations between repeated measurements within each participant. Stata version 16 was used to test the effect of MiCBT on each outcome measure using a mixed effects restricted maximum likelihood (REML) regression for each outcome variable with participants included as random effects intercepts and group and time as fixed effects.

An important advantage of the mixed model over repeated measures ANOVA is that the modeling of the individual participant variables can accommodate multiple missing data points in longitudinal datasets (55). All available data were therefore included in the analysis and, in line with the intention-to-treat approach, regardless of compliance with the treatment protocol (56). Although mixed-effects REML is robust to missing data (55), a sensitivity analysis was also conducted by imputing missing data for the primary outcome (DASS-21) using multiple-imputation by chained equations (MICE) (57).

#### Mediation Analyses

An overview of the two planned mediation models is described in Francis et al. (22, 23). The first planned model comprised: group (MiCBT vs. TAU-wait-list control) as the IV; clinical and wellbeing measures as the DVs; and awareness (metacognitive and interoceptive) and equanimity measures that revealed significant group x time interactions as mediators. It was intended to test changes occurring both during the program and during the follow-up phase. The second planned model comprised: group as the IV; T3 psychological wellbeing as the DV; and T2 social functioning, awareness and equanimity measures that revealed significant group x time interactions as mediators.

**Figure 2:**
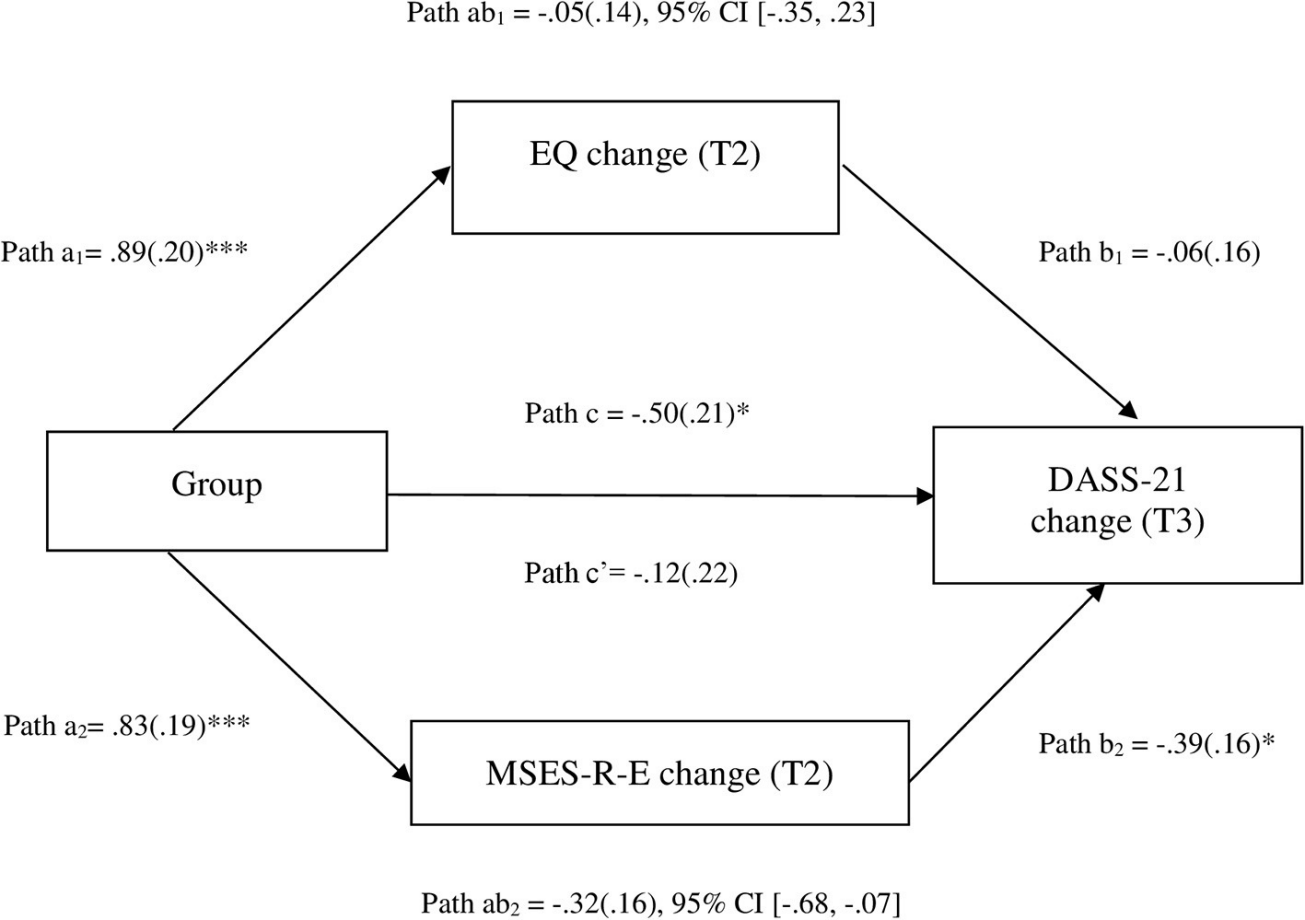
Path diagram depicting parallel mediation model 1, testing whether changes in equanimity (MSES-R-E) and awareness (EQ) mediate the effects of MiCBT vs. control on improvements in the DASS-21. Unstandardized path coefficients are displayed. * < 0.05; *** < 0.001. EQ, Experiences Questionnaire; MSES-R-E, Mindfulness-based Self-Efficacy Scale-Revised-Equanimity subscales; DASS-21, Depression, Anxiety, and Stress Scale-21. a, b, c′ and c are unstandardized regression coefficient (with standard errors) which represent predicting the mediator from group (a), DASS-21 change form M controlling for group (b), DASS-21 change from group controlling for both mediators (direct effect, c′), and DASS-21 change from group not controlling for the mediators (total effect, c). The product of a and b paths, ab, represents the mediated or indirect effect. Change refers to standardized residualized changed scores.

Data were analyzed using the Statistical Package for Social Sciences (SPSS) Version 26.0 software (58). An alpha level of .05 was set for the main inferential tests. All mediation analyses were conducted using the PROCESS macro version 3.5 for SPSS; unstandardized regression coefficients are reported (59). Path coefficients were computed in PROCESS for: path a, the effect of the IV on the mediator; path b, the effect of the mediator on the DV controlling for the IV; path c, the total effect of the IV on the DV; and path c′, the direct effect of the IV on the DV controlling for the mediator. Point estimates of each indirect effect (ab) and percentile bootstrapped confidence intervals were computed by averaging the ab product from 5,000 random samples of the original data. Indirect effects are significant if the lower and upper boundaries of the bootstrapped 95% confidence intervals (CIs) do not include zero. Figure 2 displays the a, b, c, c′ and ab paths.

Following correlation analysis of the model variables using Pearson's correlation coefficients, model building commenced with a series of single mediation models to identify relevant mediators for the final model (60, 61). To take into account changes over time, baseline (pre-intervention period) measures of the outcome and mediator (“lags”) were included in the modeling as covariates in preference to difference scores, as recommended by Hayes (59) and Valente and MacKinnon (62). Similar to Gu et al. (63), analyses for the two planned multiple mediation models used standardized residualized change scores for mediator and outcome variables calculated using linear regression in which baseline scores predicted post-intervention scores for mediators and baseline scores predicted follow-up scores for the outcome variable.

#### Exploratory Analysis: Clinically Significant Change

An exploratory analysis of clinically significant change was conducted for the DASS-21 using descriptive, chi-square and odds ratio (OR) statistics and based on an adaptation of the Ronk et al. classification system (64) for indicating recovery (deteriorated, improved, recovered). We used the following categories to define clinical significance: normal range; mild-moderate range (collapsing mild and moderate together) and severe-extreme range (collapsing severe and extreme categories together). We defined deteriorated as unimproved; making no clinically significant positive change; (i.e., did not move to a less severe category). Improved was defined as moved to a less severe range and recovered was defined as moved to normal range.

## Results

Data from 118 out of the 125 participants were analyzed (MiCBT group = 61; Control group = 57) because 7 participants were lost to the study prior to commencement. The MiCBT and control groups were compared at time points: pre- (T0), mid- (T1), post- (T2), and at 6-month follow-up (T3). Missing data sets (entire data set) across timepoints were: T0 = 8; T1 = 12; T2 = 24; T3 = 22. There were 8 incomplete data sets (respondent fatigue). The missing data pattern was non-monotone. Preliminary analysis using binary logistic regression indicated that missing data for the primary outcome variable, the DASS-21, across the four data collection points was not associated with age, gender, education, baseline K10 and medication. We therefore assumed the data was missing at random.

There were no significant differences in K10 change scores from referral to baseline between the control group (*M* = −0.28, *SD* = 5.58) and MiCBT group [*M* = −2.00, *SD* = 5.64; *t*(116) = 1.66, *p* = 0.10, two-tailed]. Although more participants in the MiCBT group reported a reduction in K10 symptoms in the waiting period before the program commenced the mean difference = 1.72 (95% CI: −0.33 to 3.77) was small (eta squared = 0.02).

The mean number of meditation practice hours across the 8-week program was 27.25 (13.70) median = 30 h; range = 0–49. The mean weekly practice hours were 4.47 h. The mean number of classes attended was 6.22 (1.87); median 7.0; range = 1–8. The dropout rate from data collection at the final assessment point for the MiCBT and control groups respectively was 22% (14/64) and 25% (15/61), respectively. Excluding participants who were lost to the study before completing the baseline assessment, the dropout rate from MiCBT treatment, defined as insufficient treatment (<4 sessions), was 23.0% (14/61). Information about medications at 6-month follow up was collected from the MiCBT group and 82% (37/45) reported no change to their medications while 4 participants lowered or ceased medications; 3 changed medications (no details provided) and 1 participant increased the medication dose.

In relation to adverse effects, 19 (31%) of the MiCBT participants responded “yes” to the question, “Did you have any unexpected, challenging or difficult experiences that you associate with your practice of meditation?” Of these, 12 participants provided a description of their experience in response to the question, “How did these experiences impact your life” (see Table 3 for descriptions).

**Table 3:** Unexpected challenging or difficult effects associated with meditation.

**Responses that described effects that may be considered adverse (*n* = 12)**
1. They caused mood swings and changed the way some of my relationships progressed.	2. I needed to rest a lot.	3. Limited time to practice.	4. Not to such a great extent but I can become irritable.	5. Some positive, some negative.	6. Hard to explain but affected my anxiety.	7. Made me want to avoid meditating.	8. In the beginning there were extremely intense sensations that were really, really distracting, particularly at work. I spent my first week constantly feeling as though I would have a panic attack, along with intense pain throughout my body. I had to remind myself it was just part of the process. With a difficult first week and a few moments of intense memories throughout body scanning, the results were incredible. I never anticipated to feel “at peace”, or “content” in my life… I think that's getting closer to happiness? Relationships in my life have improved dramatically, and the ruminating over this person and that person, this situation or that, wasn't an issue—it's like a happy oblivion, but still aware of all the craziness.	9. They brought unprocessed trauma closer to the surface.	10. The body scanning brought up some issues that I had placed on emotional lockdown and I went through a period of intense healing and rest after doing the course.	11. For the first 2 weeks meditation felt great, around 4–6 weeks I noticed that I was becoming more aware of challenging emotions in my body, that were perhaps previously ignored.	12. Some PTSD triggers.

### Main Analysis

Supplementary Table 1 presents the means, standard deviations, and medians of all measurement instruments at the four time points for each group. Supplementary Table 2 presents the means, standard deviations, and medians of the three subscales of the DASS-21. Table 4 and Supplementary Table 3 presents the intervention differences estimated by the regression model for the effects of time (i.e., model adjusted intervention difference) for the outcome and mediator measures, respectively, and Table 5 presents the effect sizes. Timepoints T1–T3 are all compared to baseline T0. Intervention status is MiCBT compared to control. Results showed that all outcome and mediation measures showed significant improvement over time compared to baseline.

**Table 4:** Mixed REML regression models for outcome variables with fixed factors of time and intervention status, and participants as random.

**DASS-21**	***b***	**se**	***z***	***p-*value**	**95%Conf**.	**Interval**
**Timepoint**							
T1	−7.46	2.13	−3.51	0.000	−11.62	−3.29
T2	−12.08	2.54	−4.75	0.000	−17.06	−7.09
T3	−10.38	2.17	−4.78	0.000	−14.63	−6.13
**Intervention status**							
1	−9.17	1.61	−5.69	0.000	−12.33	−6.01
Number of obs = 413, Number of groups = 118, Bootstrap replications = 50						
Log restricted-likelihood = −1,791.41, Wald chi^2^ (4) = 73.89, Prob > chi^2^ = 0.00.						
**K10**	***b***	**se**	***z***	***p-*** **value**	**95%Conf**.	**Interval**
**Timepoint**							
T1	−3.60	0.78	−4.63	0.000	−5.13	−2.08
T2	−4.42	0.80	−5.51	0.000	−6.00	−2.85
T3	−4.79	0.86	−5.57	0.000	−6.48	−3.11
**Intervention status**							
1	−3.54	0.55	−6.48	0.000	−4.62	−2.47
Number of obs = 405, Number of groups = 118, Bootstrap replications = 50,						
Log restricted-likelihood = −1,307.54, Wald chi^2^ (4) = 97.97, Prob > chi^2^ = 0.00						
**SWLS**	***b***	**se**	***z***	***p-*** **value**	**95%Conf**.	**Interval**
**Timepoint**							
T1	2.12	0.60	3.50	0.000	0.93	3.30
T2	2.96	0.57	5.22	0.000	1.85	4.07
T3	3.08	0.53	5.78	0.000	2.03	4.12
**Intervention status**							
1	0.59	0.44	1.35	0.177	−0.27	1.45
Number of obs = 411, Number of groups = 118, Bootstrap replications = 50,						
Log restricted-likelihood = −1,245.15, Wald chi^2^ (4) = 47.31, Prob > chi^2^ = 0.00.						
**FS**	***b***	**se**	***z***	***p-*** **value**	**95%Conf**.	**Interval**
**Timepoint**							
T1	2.86	0.63	4.52	0.000	1.62	4.10
T2	3.84	0.74	5.17	0.000	2.39	5.30
T3	4.26	0.75	5.70	0.000	2.79	5.72
**Intervention status**							
1	2.17	0.58	3.74	0.000	1.03	3.30
Number of obs = 411, Number of groups = 118, Bootstrap replications = 50,						
Log restricted-likelihood = −1,333.32, Wald chi^2^ (4) = 73.53, Prob > chi^2^ = 0.00.						

*DASS-21, Depression, Anxiety, and Stress Scale-21; K10, Kessler Psychological Distress Scale; SWLS, Satisfaction with Life Scale; FS, Flourishing Scale*.

**Table 5:** Means and effect sizes from mixed REML regression models for outcome and mediator variables.

	**Model calculated (95% CI)**			**Estimated** **Cohen's d**
	**Control**	**Intervention**	**Difference**	
**Outcome variables**				
DASS-21	47.12 (44.97, 49.27)	37.95 (35.82, 40.08)	−9.17 (−12.33, −6.01)	0.38
K10	27.19 (26.43, 27.95)	23.65 (22.96, 24.34)	−3.54 (−4.62, −2.47)	0.45
SWLS	18.95 (18.36, 19.54)	19.55 (18.98, 20.11)	0.59 (−0.27, 1.45)	0.08
FS	37.35 (36.52, 38.18)	39.51 (38.78, 40.24)	2.17 (1.03, 3.30)	0.24
**Mediator variables**				
MAIA	62.38 (61.02, 63.73)	74.61 (72.81, 76.40)	12.23 (10.09, 14.38)	0.67
NAS	108.41 (106.62, 110.22)	119.89 (117.70, 122.07)	11.47 (8.73, 14.21)	0.48
EQ (decentering)	30.10 (29.57, 30.64)	33.93 (33.24, 34.62)	3.83 (2.95, 4.71)	0.16
MSES-R-E (Equanimity)	26.29 (25.67, 26.92)	30.09 (29.21, 30.97)	3.80 (2.71, 4.88)	0.50
MSES-R-S (Interpersonal)	22.28 (21.78, 22.77)	24.07 (23.56, 24.58)	1.79 (1.07, 2.51)	0.34

*DASS-21, Depression, Anxiety, and Stress Scale-21; K10, Kessler Psychological Distress Scale; SWLS, Satisfaction with Life Scale; FS, Flourishing Scale; MAIA, Multi-dimensional Assessment of Interoceptive Awareness; NAS, Non-attachment Scale; EQ, Experiences Questionnaire; MSES-R-E, Mindfulness-based Self-Efficacy Scale-Revised-Equanimity subscales; MSES-R-S, Mindfulness-based Self-Efficacy Scale-Revised-Interpersonal skills subscales*.

The primary outcome measure (DASS-21) demonstrated significant differences between the MiCBT and the control groups (*p* < 0.0001) as did the K10 and the FS. Only the SWLS failed to reach significance (*p* = 0.177). The differences between the MiCBT and control groups adjusting for the effects of time were as follows: DASS-21 −9.17 (95% CI: −12.33 to −6.01); K10 −3.54 (95% CI: −4.62 to −2.47); SWLS 0.59 (95% CI: −0.27 to 1.45); FS 2.17 (95% CI: 1.03 to 3.30). All mediation measures demonstrated significance (*p* < 0.0001). The differences between the MiCBT and control groups adjusting for the effects of time were as follows: MSES-R-E (Equanimity) 3.80 (95% CI: 2.71 to 4.88); MSES-R-S (Interpersonal skills) 1.79 (95% CI: 1.07 to 2.51); MAIA 12.23 (95% CI: 10.09 to 14.38); NAS 11.47 (95% CI: 8.73 to 14.21); EQ 3.83 (95% CI: 2.95 to 4.71).

The results of sensitivity analysis for the DASS-21 using MICE to impute the missing data remained significant with the coefficients changing only marginally (see Supplementary Table 4).

Because all the measures at baseline were similar in both groups (Supplementary Table 1), and improved at each time point following baseline, the intervention status results provide evidence that MiCBT improved participant outcomes on the DASS-21, K10, and FS compared to controls along with all mediation measures.

### Mediation Analyses

Correlations between model variables are shown in Supplementary Tables 5, 6. T0 correlations were substantially lower than at T3 and therefore the strength of correlation changed as a function of the intervention. Because all mediators showed significant group × time interactions in the main analyses, we constrained the number of analyses by limiting the DV to the primary outcome (rather than including both the DASS-21 and the K10), and the time frame to the follow-up (rather than both the follow-up and treatment period). Focusing on mediators measured at the end of treatment affecting outcome measured at 6-month follow-up enabled the examination of mechanisms related to the full effects of treatment and their sustainability over time, which we considered to be of most interest. This timeframe allowed for changes in the mediator during the treatment phase to be measured prior to the change in outcome.

The single mediation analyses included four independent variable mediation models (MAIA, EQ, NAS, MSES-R-E) to assess symptom change in the DASS-21 as a function of MiCBT vs. control during the timeframe of the follow-up period (mediators measured at post-intervention; outcomes at 6 months) and five independent variable mediation models (MAIA, EQ, NAS, MSES-R-E, MSES-R-S) assessing change in the FS as a function of treatment condition during the same timeframe. The MAIA, EQ, NAS, and MSES-E were each significant mediators between treatment and outcome as measured by DASS-21 (see Supplementary Table 7). In addition, the MAIA, EQ, NAS and MSES-E, and MSES-S were significant mediators between intervention and outcome as assessed by FS (see Supplementary Table 8).

#### Model 1

Awareness (interoceptive awareness measured by the MAIA and metacognitive awareness measured by EQ) and equanimity (measured by the MSES-R-E and NAS) are the core focus in MiCBT, which are developed independently and simultaneously (65). The relative influence of awareness and equanimity on outcomes was examined in the first (parallel) multiple mediation model. Given theoretical overlap and large correlations between the mediators (*r* = 0.45–0.85) one candidate variable was selected from each construct based on having the strongest correlation with the DASS-21 at time 3 (see Supplementary Table 6). These were MSES-R-E and EQ. Results are shown in Figure 2. While 65.3% of the total effect of MiCBT relative to control on the DASS-21 at time 3 was due to the indirect effect of MSES-R-E measured at time 2, only 10.8% of total effect was due to the indirect effect of EQ at time 2. While only MSES-R-E had a significant indirect effect on the DASS-21 at time 3, the difference between the two indirect effects was not significant. See Supplementary Table 9 for the PROCESS macro version 3.5 for SPSS output for model 1.

#### Model 2

In a second parallel model (Supplementary Figure 1), we examined whether the effect of MiCBT vs. control on flourishing scale scores are mediated more by improvements in interpersonal skills compared to awareness and equanimity. While the total indirect effect of EQ, MSES-R-E and MSES-R-S was significant, *B* = 0.43 (0.15) 95% CI (0.19 to 0.75), and accounted for 64.8% of the total effect of Group on FS at time 3, none of the individual mediators when combined had a significant indirect effect. See Supplementary Table 10 for the PROCESS macro version 3.5 for SPSS output for model 2.

### Exploratory Analysis: Clinically Significant Change

In the control group, 40.5% (17/42) of participants were unimproved based on DASS-21 scores and 21.4% (9/42) were recovered at follow up. Participants in the MiCBT group showed significantly greater clinical improvement compared to controls with just 16.7% (8/48) unimproved and 41.7% (20/48) recovering. Overall, at 6-month follow-up 83.3% (40/48) of participants in the MiCBT group showed improvement or recovery compared to 59.5% (25/42) of participants in the control group. Chi-square for group differences was 6.3, df = 1, *p* = 0.01 (*n* = 90). Individuals in the MiCBT group had higher odds of a successful treatment (OR: 3.4, 95% CI: 1.3 to 9.0; *p* = 0.01) and their relative risk for deterioration was 0.4 (95% CI: 0.2 to 0.9).

In the MiCBT group, 74% of participants who improved or recovered did at least 20 h of meditation practice (i.e., 2.5 h per week) while 26% improved or recovered with <20 h. Fifty-two percent of those who recovered or improved attended all 8 sessions and 94% of improved/recovered group attended at least 5 sessions.

Further exploratory analysis of the data from those who had severe levels of distress at T0 suggests that the MiCBT intervention was effective for severe anxiety with 77% recovered or improved (13/17) compared to 33% (4/12) recovered or improved in the control group. For those with severe levels of stress at T0, 81% recovered or improved (13/19) compared to 40% (6/15) in the control group. MiCBT was no better than usual treatment in the severely depressed group (50%−5/10 improved or recovered) compared to 57% (8/14) of the control group.

## Discussion

In the present study we investigated the effectiveness of MiCBT as a transdiagnostic intervention delivered in a naturalistic primary health care setting. The findings support our hypotheses that MiCBT would (a) reduce levels of depression, anxiety and stress as measured by the total DASS-21 scores and (b) improve flourishing significantly more than TAU, and (c) that such differential improvements would be sustained over a 6-month follow-up period. The findings did not, however, support our hypothesis of improved life satisfaction—although scores trended toward improvement, the change did not reach significance.

Results of parallel mediation models suggested that equanimity was the most important change variable for symptom reduction and interpersonal skills did not show a significant mediation effect for flourishing when compared to awareness (decentering) and equanimity. That equanimity was the most important change variable is consistent with the theoretical framework provided by the co-emergence model of reinforcement (24). Specifically, the acquisition of equanimity is facilitated by scanning the body systematically in detail without reacting to perceived body sensations.

The range of severity of clinical symptoms (mild to severe) was reflective of presentations in primary health care in Australia where a K10 score >20 is frequently used to initiate treatment referral. The rate of adherence to the MiCBT treatment is consistent with other studies [e.g., (66, 67)] with 77% (47/61) of participants receiving four or more sessions. The mean practice hours was less than the recommended 7 h per week (64% of recommended). This is consistent with a study of practice time in a Mindfulness-based Cognitive therapy (MBCT) program (68) which reported that participants who were asked to practice 6 days out of seven in fact practiced on average on 3.36 days (56% of recommended) per week. In the current study, adherence to standard program delivery was supported using audio instructions that provide both a rationale for each exercise, and guidance for each meditation type. In this way participants have some of the CBT elements of the rationale reinforced.

A strength of the current study is that data about so-called adverse effects was actively collected. The data indicated that 19 participants (31%) responded “yes” to the question on “unexpected, challenging, or difficult experiences that you associate with your practice of meditation”. However, only 12 participants provided any detailed descriptive responses. Six of the twelve descriptions were vague (Table 3 responses 2–7); one response described mood changes and four reported that past trauma was triggered. One response while describing initially difficult experiences went on to describe very positive results “With a difficult first week and a few moments of intense memories throughout body scanning, the results were incredible…” (Table 3 response 8). In MiCBT, trainees are explicitly forewarned that both pleasant and unpleasant body sensations are to be expected during practice, and that they are to remain equanimous to whatever arises.

Different frequencies of adverse effects from meditation in a mindfulness-based intervention (MBI) have been reported dependent on methodological approach ranging from 3.7 to 33.2% (60). Furthermore, it is recognized that in the Buddhist Vipassana tradition there are stages of development of insight or knowledge, some of which involve negative experiences such as fear, sense of dissolution, disgust, which may result in potential clinical effects (69). Challenges in MiCBT are to be expected because the therapeutic strategy is fundamentally exposure based: by developing equanimity toward sensations, a desensitization process takes place, enabling tolerance of previously intolerable experience. For many people the experience of physical pain and psychological distress is deemed intolerable, and pharmaceutical pain-relieving interventions are sought. Consequently, instruction to sit with discomfort non-reactively may be very challenging for some people, especially when the level of equanimity is not yet well developed. Interestingly, a recent study demonstrated that there was no relationship between the occurrence of an emotional experience early in a mindfulness intervention and adverse outcomes or drop-out rate (70). In some instances, participants clearly understood that any difficulties were a natural part of the process, and that the development of equanimity enabled a transformation of attitude to difficulties (e.g., Table 3, response 8). Data on unexpected or unpleasant effects was only collected at follow-up which may be a limitation of the study. While most of those who discontinued the program provided reasons such as changed personal circumstances, it is possible that some people dropped out due to unpleasant effects. The dropout rate was consistent with other studies which reported between 14 and 25% drop out rates (71).

Results indicate significant positive change across most measures both clinical and non-clinical after only 4 weeks of the program. There was further symptom reduction at week 8 and at follow up the changes were mostly maintained or enhanced. That the pattern of change was consistent across all measures is of interest because the interpersonal skills are not explicitly taught until the second half of the program. It appears that the elements of stage 1 of the intervention generalized across both clinical and non-clinical measures. These results may indicate the importance of the co-emergence model of reinforcement in providing a sound cognitive rationale for engaging in the meditation elements, body scanning in particular. The co-emergence model is used as a psycho-education tool to assist participants to understand the relationship between evaluative thinking and co-arising body sensations. Using the model may be instrumental in motivating participants to complete the daily meditation tasks.

### Limitations and Future Research

This study has several limitations. Firstly, the lack of an active control precludes being able to distinguish between improvements that may be due to generic group processes as opposed to the MiCBT skill acquisition. However, the findings from the mediation analysis provide some evidence that the effects of MiCBT operated through its hypothesized mechanisms, especially equanimity. Nonetheless, to build the evidence for the specific effects of MiCBT, future research could test the comparative effectiveness of MiCBT against an active control such as CBT, MBCT or other third wave therapies.

Secondly, the sample was not representative of the general Australian population. Specifically, there was low representation of participants born outside Australia (12%) compared to 30% in the general population and our sample was also more highly educated than the general population with over 80% of participants with post-school education compared to 56% for the general population (72). It is possible that the population willing to engage in meditation-based therapies tend to be more educated as there is some requirement for homework and effortful meditations.

Thirdly, while we collected information on medications, we did not collect data on any other therapeutic interventions that participants may have been offered. Also, although we asked MiCBT group participants at follow up if there had been any changes to medications, we did not collect this data from the control group. Future studies might investigate the possibility of reductions of medication needs in patients whose depression, anxiety and stress scores improve following the MiCBT intervention.

Fourthly, the same therapist (the first author) conducted all the group training limiting generalizability. Future research could replicate the study with different facilitators including, potentially, at different skill levels as this has implications for treatment dissemination (73). The use of the most recent audio instructions recorded by the developer of the program (BC) would support consistency in delivery, as mentioned earlier. The current study also used the 8-week protocol (10) that was available at the start of the study. The protocol has since been extended to a 10-week protocol (11) and future studies might investigate results with the more recent protocol.

Fifthly, the baseline measures for the study were administered post-randomization, 1 week prior to the start of the program. While this had the advantage of accounting for the delay in recruitment for some groups to maximize sensitivity to change in the context of fluctuating mental health conditions, it also had the potential to produce a systematic observer or participant bias. However, we consider this to be unlikely. Observer bias is unlikely because the main measures were administered online. Participant bias is still possible but perhaps minimal because there were no significant group differences between the (pre-randomization) medical practitioner administered K10 and the (post-randomization) baseline K10 that would suggest, for example, demoralization in the control group. A disadvantage was that seven participants were lost to the study post-randomization while awaiting the start date. All participants were aware from the commencement of the study that they would be offered MiCBT within a reasonable time frame.

Sixthly, the exploratory analysis examining clinically significant change applied the severity cut-off scores from the DASS manual. These were included by the manual's authors to help characterize the severity of symptoms relative to the population. On the manual website, http://www2.psy.unsw.edu.au/dass//DASSFAQ.htm#_5._ it is noted that emotional syndromes such as anxiety are dimensional, and the use of cut-offs are necessarily arbitrary and should not be reified. However, as we are assessing change pre- and post-treatment in an Australian sample, these cut-offs provide a potentially useful indication of clinical effects since these cut-off scores are widely used clinically in this country.

A final limitation of the study to note is that it was designed using K10 scores as inclusion criteria rather than diagnosis which would have better supported the transdiagnostic applicability of MiCBT.

Notwithstanding these limitations, the present results add to previous research supporting to the effectiveness of the MiCBT group-based intervention. The use of a randomized controlled trial in a naturalistic private psychology clinic setting with a heterogenous population is a contribution to this under-researched area. The collection of data at the mid-point as well as at 8 weeks and 6-month follow up provides useful information about timelines for change. Importantly, the results support the transdiagnostic applicability of MiCBT, demonstrating significant improvements in clinical outcomes in the first 4 weeks of treatment, increasing in the second half of the program and being maintained over a 6-month period across a population with a range of disorders and levels of distress severity. It is noteworthy that significant gains were apparent after just 4 weeks despite the amount of meditation practice undertaken by participants being less than prescribed. Future studies could explore what may be minimum practice requirements while still achieving clinical improvements and whether the gains at 4 weeks can be maintained even without receiving the remaining 4 weeks of the program or whether the second half of the program is essential for long-term change.

## Data Availability Statement

The dataset generated for this study can be found in the research repository of Monash University: https://www.monash.edu/library/researchers/researchdata/bridges: DOI: 10.26180/13240304.

## Ethics Statement

This study involving human participants was reviewed and approved by Monash University Human Research Ethics Committee (Project Number: CF16/2278 - 2016001131). The participants provided their written informed consent to participate in this study.

## Author Contributions

SF conducted the research and led the writing of this paper as part of a PhD thesis. FS was the primary supervisor and GM and BC were co-supervisors. JE and FS devised and guided the statistical analyses. FS, GM, and BC contributed to the writing, editing, and critical revision of the intellectual content of the manuscript. All authors approved the final version of this manuscript.

## Conflict of Interest

BC and SF have been paid for developing and delivering educational presentations, workshops and professional training programs for the MiCBT Institute in Australia and North America. BC and SF receive royalties for the text: *The Clinical Handbook of Mindfulness-integrated Cognitive Behavior Therapy: A Step-by-step Guide for Therapists*. The remaining authors declare that the research was conducted in the absence of any commercial or financial relationships that could be construed as a potential conflict of interest.

## Publisher's Note

All claims expressed in this article are solely those of the authors and do not necessarily represent those of their affiliated organizations, or those of the publisher, the editors and the reviewers. Any product that may be evaluated in this article, or claim that may be made by its manufacturer, is not guaranteed or endorsed by the publisher.

## References

[1] Kessler RC Andrews G Colpe LJ Hiripi E Mroczek DK Normand S-L. Short screening scales to monitor population prevalences and trends in non-specific psychological distress. Psychol Med. (2002) 32:959–76. 10.1017/S0033291702006074 12214795

[2] Pollack MH. Comorbid anxiety and depression. J Clin Psychiatry. (2005) 66(Suppl. 8):22. 16336033

[3] Sekula LK DeSantis J Gianetti V. Considerations in the management of the patient with comorbid depression and anxiety. J Am Acad Nurse Pract. (2003) 15:23–33. 10.1111/j.1745-7599.2003.tb00251.x 12613410

[4] Chen L-Y Crum RM Martins SS Kaufmann CN Strain EC Mojtabai R. Service use and barriers to mental health care among adults with major depression and comorbid substance dependence. Psychiatric Services. (2013) 64:863–70. 10.1176/appi.ps.201200289 23728427 PMC4049190

[5] Harvey AG Watkins E Mansell W. Cognitive Behavioural Processes Across Psychological Disorders: A Transdiagnostic Approach to Research and Treatment. Oxford: Oxford University Press (2004).

[6] Watkins E. An alternative transdiagnostic mechanistic approach to affective disorders illustrated with research from clinical psychology. Emotion Rev. (2015) 7:250–5. 10.1177/1754073915575400

[7] Ehring T Watkins ER. Repetitive negative thinking as a transdiagnostic process. Int J Cogn Ther. (2008) 1:192–205. 10.1521/ijct.2008.1.3.19230851652

[8] Barlow DH Allen LB Choate ML. Toward a unified treatment for emotional disorders. Behav Ther. (2004) 35:205–30. 10.1016/S0005-7894(04)80036-4 27993336

[9] Norton PJ Paulus DJ. Toward a unified treatment for emotional disorders: update on the science and practice. Behav Ther. (2016) 47:854–68. 10.1016/j.beth.2015.07.002 27993337

[10] Cayoun BA. Mindfulness-Integrated CBT: Principles and Practice. New York, NY: John Wiley & Sons (2011).

[11] Cayoun BA. Mindfulness-Integrated Cbt For Well-Being And Personal Growth : Four Steps To Enhance Inner Calm, Self-Confidence And Relationships. Chichester, WS: Wiley/Blackwell (2015). vi, 292 p.

[12] Cayoun BA Francis S Shires AG. The Clinical Handbook of Mindfulness-integrated Cognitive Behavior Therapy: A Step-by-step Guide for Therapists. John Wiley & Sons (2019).

[13] Fernandez KC Jazaieri H Gross JJ. Emotion regulation: a transdiagnostic perspective on a new RDoC domain. Cognit Ther Res. (2016) 40:426–40. 10.1007/s10608-016-9772-2 27524846 PMC4979607

[14] Desbordes G Gard T Hoge EA Hölzel BK Kerr C Lazar SW. Moving beyond mindfulness: defining equanimity as an outcome measure in meditation and contemplative research. Mindfulness. (2015) 6:356–72. 10.1007/s12671-013-0269-8 25750687 PMC4350240

[15] Garfinkel SN Critchley HD. Interoception, emotion and brain: new insights link internal physiology to social behaviour. Soc Cogn Affect Neurosci. (2013) 8:231–4. 10.1093/scan/nss140 23482658 PMC3594730

[16] Seth AK Critchley HD. Extending predictive processing to the body: emotion as interoceptive inference. Behav Brain Sci. (2013) 36:227–8. 10.1017/S0140525X12002270 23663284

[17] Murphy J Brewer R Catmur C Bird G. Interoception and psychopathology: a developmental neuroscience perspective. Dev Cogn Neurosci. (2017) 23:45–56. 10.1016/j.dcn.2016.12.006 28081519 PMC6987654

[18] Hartley CA Phelps EA. Changing fear: the neurocircuitry of emotion regulation. Neuropsychopharmacology. (2010) 35:136–46. 10.1038/npp.2009.121 19710632 PMC3055445

[19] Khalsa SS Adolphs R Cameron OG Critchley HD Davenport PW Feinstein JS. Interoception and mental health: a roadmap. Biol Psychiatry Cogn Neurosci Neuroimag. (2018) 3:501–13. 10.1016/j.bpsc.2017.12.004 29884281 PMC6054486

[20] Barrett LF Gross J Christensen TC Benvenuto M. Knowing what you're feeling and knowing what to do about it: mapping the relation between emotion differentiation and emotion regulation. Cogn Emot. (2001) 15:713–24. 10.1080/02699930143000239

[21] Vøllestad J Sivertsen B Nielsen GH. Mindfulness-based stress reduction for patients with anxiety disorders: evaluation in a randomized controlled trial. Behav Res Ther. (2011) 49:281–8. 10.1016/j.brat.2011.01.007 21320700

[22] Francis S Shawyer F Cayoun B Enticott J Meadows G. Study protocol for a randomized control trial to investigate the effectiveness of an 8-week mindfulness-integrated cognitive behavior therapy (MiCBT) transdiagnostic group intervention for primary care patients. BMC Psychiatry. (2020) 20:7. 10.1186/s12888-019-2411-1 31906903 PMC6945698

[23] Francis S Shawyer F Cayoun B Enticott J Meadows G. Correction to: study protocol for a randomized control trial to investigate the effectiveness of an 8-week mindfulness-integrated cognitive behavior therapy (MiCBT) transdiagnostic group intervention for primary care patients. BMC Psychiatry. (2020) 20:136. 10.1186/s12888-020-02534-y 32216784 PMC7099767

[24] Cayoun BA Shires AG. Co-emergence reinforcement and its relevance to interoceptive desensitization in mindfulness and therapies aiming at transdiagnostic efficacy. Front. Psychol. (2020) 11:e545945. 10.3389/fpsyg.2020.545945 33414739 PMC7783049

[25] Barrett LF. Valence is a basic building block of emotional life. J Res Pers. (2006) 40:35–55. 10.1016/j.jrp.2005.08.006

[26] Barrett LF. The theory of constructed emotion: an active inference account of interoception and categorization. Soc Cogn Affect Neurosci. (2017) 12:1–23. 10.1093/scan/nsx060 27798257 PMC5390700

[27] Eraslan-Capan B. Social connectedness and flourishing: the mediating role of hopelessness. Univ J Educ Res. (2016) 4:933–40. 10.13189/ujer.2016.040501

[28] Cayoun BA. The purpose, mechanisms and benefits of cultivating ethics in Mindfulness-integrated Cognitive Behavior Therapy. In: Monteiro CM, editor. Practitioner's Guide to Ethics and Mindfulness-Based Interventions. Cham: Springer (2017). p. 163–92.

[29] Bahrani S Zargar F Yousefipour G Akbari H. The effectiveness of mindfulness-integrated cognitive behavior therapy on depression, anxiety, and stress in females with multiple sclerosis: a single blind randomized controlled trial. Iranian Red Crescent Med J. (2017) 19:466. 10.5812/ircmj.44566

[30] Pouyanfard S Mohammadpour M ParviziFard AA Sadeghi K. Effectiveness of mindfulness-integrated cognitive behavior therapy on anxiety, depression and hope in multiple sclerosis patients: a randomized clinical trial. Trends Psychiatry Psychother. (2020) 42:55–63. 10.1590/2237-6089-2018-0105 32321085

[31] Yazdanimehr R Omidi A Sadat Z Akbari H. The effect of mindfulness-integrated cognitive behavior therapy on depression and anxiety among pregnant women: a randomized clinical trial. J Caring Sci. (2016) 5:195. 10.15171/jcs.2016.021 27752485 PMC5045953

[32] Scott-Hamilton J Schutte NS Brown RF. Effects of a mindfulness intervention on sports-anxiety, pessimism, and flow in competitive cyclists. Appl Psychol Health Well-ellho. (2016) 8:85–103. 10.1111/aphw.12063 26970111

[33] Mozafari-Motlagh M-R Nejat H Tozandehjani H Samari A-A. Effect of cognitive behavior therapy integrated with mindfulness on perceived pain and pain self-efficacy in patients with breast cancer. J Nurs Midwifery Sci. (2019) 6:51. 10.4103/JNMS.JNMS_60_18

[34] Cayoun BA Simmons A Shires A. Immediate and lasting chronic pain reduction following a brief self-implemented mindfulness-based interoceptive exposure task: a pilot study. Mindfulness. (2020) 11:112–24. 10.1007/s12671-017-0823-x

[35] Wang L Zhang M Zhu H Sun L Yu B Cui X. Combined identification of lncRNA NONHSAG004550 and NONHSAT125420 as a potential diagnostic biomarker of perinatal depression. J Clin Lab Anal. (2021) 35:e23890. 10.1002/jcla.23890 34263944 PMC8373316

[36] Francis S. A preliminary study of mindfulness-integrated cognitive behaviour therapy: results from a series of group interventions. In: First International Conference on Mindfulnes.; Rome, Italy (2013).

[37] Eisendrath SJ Gillung EP Delucchi KL Chartier M Mathalon DH Sullivan JC. Mindfulness-based cognitive therapy (MBCT) versus the health-enhancement program (HEP) for adults with treatment-resistant depression: a randomized control trial study protocol. BMC Complement Altern Med. (2014) 14:95. 10.1186/1472-6882-14-95 24612825 PMC3995768

[38] Fritz MS Mackinnon DP. Required sample size to detect the mediated effect. Psychol Sci. (2007) 18:233–9. 10.1111/j.1467-9280.2007.01882.x 17444920 PMC2843527

[39] Lovibond PF Lovibond SH. The structure of negative emotional states: comparison of the Depression Anxiety Stress Scales (DASS) with the Beck Depression and Anxiety Inventories. Behav Res Ther. (1995) 33:335–43. 10.1016/0005-7967(94)00075-U 7726811

[40] Henry JD Crawford JR. The short-form version of the Depression Anxiety Stress Scales (DASS-21): construct validity and normative data in a large non-clinical sample. Br J Clin Psychol. (2005) 44:227–39. 10.1348/014466505X29657 16004657

[41] Lovibond SH Lovibond PF. Manual for the Depression Anxiety Stress Scales. 2nd ed. Sydney, NSW: Psychology Foundation (1995).

[42] Andrews G Slade T. Interpreting scores on the Kessler psychological distress scale (K10). Aust N Z J Public Health. (2001) 25:494–7. 10.1111/j.1467-842X.2001.tb00310.x 11824981

[43] Furukawa TA Kessler RC Slade T Andrews G. The performance of the K6 and K10 screening scales for psychological distress in the Australian National Survey of Mental Health and Well-Being. Psychol Med. (2003) 33:357–62. 10.1017/S0033291702006700 12622315

[44] Diener E Emmons RA Larsen RJ Griffin S. The satisfaction with life scale. J Pers Assess. (1985) 49:71–5. 10.1207/s15327752jpa4901_13 16367493

[45] Mehling WE Price C Daubenmier JJ Acree M Bartmess E Stewart A. The Multidimensional assessment of interoceptive awareness (MAIA). PLoS ONE. (2012) 7:e48230. 10.1371/journal.pone.0048230 23133619 PMC3486814

[46] Sahdra BK Shaver PR Brown KW. A scale to measure nonattachment: a Buddhist complement to Western research on attachment and adaptive functioning. J Pers Assess. (2010) 92:116–27. 10.1080/00223890903425960 20155561

[47] Fresco DM Moore MT van Dulmen MH Segal ZV Ma SH Teasdale J. Initial psychometric properties of the experiences questionnaire: validation of a self-report measure of decentering. Behav Ther. (2007) 38:234–46. 10.1016/j.beth.2006.08.003 17697849

[48] Bernstein A Hadash Y Lichtash Y Tanay G Shepherd K Fresco DM. Decentering and related constructs: a critical review and metacognitive processes model. Persp Psychol Sci. (2015) 10:599–617. 10.1177/1745691615594577 26385999 PMC5103165

[49] Fresco DM Roy AK Adelsberg S Seeley S García-Lesy E Liston C. Distinct functional connectivities predict clinical response with emotion regulation therapy. Front Human Neurosci. (2017) 11:86. 10.3389/fnhum.2017.00086 28316567 PMC5334508

[50] Wells A. Cognition about cognition: metacognitive therapy and change in generalized anxiety disorder and social phobia. Cogn Behav Pract. (2007) 14:18–25. 10.1016/j.cbpra.2006.01.005

[51] Soler J Franquesa A Feliu-Soler A Cebolla A García-Campayo J Tejedor R. Assessing decentering: validation, psychometric properties, and clinical usefulness of the Experiences Questionnaire in a Spanish sample. Behav Ther. (2014) 45:863–71. 10.1016/j.beth.2014.05.004 25311294

[52] Cayoun B Elphinstone B Kasselis N Bilsborrow G Skilbeck C. Validation and factor structure of the mindfulness-based self efficacy scale-revised. Mindfulness. (2022) 13:751–65. 10.1007/s12671-022-01834-6

[53] Lindahl JR Fisher NE Cooper DJ Rosen RK Britton WB. The varieties of contemplative experience: a mixed-methods study of meditation-related challenges in Western Buddhists. PLoS ONE. (2017) 12:e0176239. 10.1371/journal.pone.0176239 28542181 PMC5443484

[54] Australian Government. National Standards for Mental Health Services 2010. Canberra, ACT: Commonwealth of Australia (2010).

[55] Krueger C Tian L. A comparison of the general linear mixed model and repeated measures ANOVA using a dataset with multiple missing data points. Biol Res Nurs. (2004) 6:151–7. 15388912 10.1177/1099800404267682

[56] Moher D Hopewell S Schulz KF Montori V Gøtzsche PC Devereaux PJ. CONSORT 2010 explanation and elaboration: updated guidelines for reporting parallel group randomised trials. BMJ. (2010) 340:c869. 10.1136/bmj.c869 20332511 PMC2844943

[57] Jakobsen JC Gluud C Wetterslev J Winkel P. When and how should multiple imputation be used for handling missing data in randomised clinical trials ria practical guide with flowcharts. BMC Med Res Methodol. (2017) 17:162. 10.1186/s12874-017-0442-1 29207961 PMC5717805

[58] IBM CORP. IBM SPSS Statistics for Windows, Version 26.0. Corp I, editor. Armonk, NY: IBM Corp. (2019).

[59] Hayes AF. Introduction to Mediation, Moderation, and Conditional Process Analysis: A Regression-based Approach. 2nd ed. New York, NY: Guilford Publications (2018).

[60] MacKinnon DP Luecken LJ. Statistical analysis for identifying mediating variables in public health dentistry interventions. J Public Health Dent. (2011) 71:S37–46. 10.1111/j.1752-7325.2011.00252.x 21656950 PMC3366631

[61] van Kesteren E-J Oberski DL. Exploratory mediation analysis with many potential mediators. Struct Equat Model Multidiscip J. (2019) 26:710–23. 10.1080/10705511.2019.1588124

[62] Valente MJ MacKinnon DP. Comparing models of change to estimate the mediated effect in the pretest–posttest control group design. Struct Equat Model Multidiscip J. (2017) 24:428–50. 10.1080/10705511.2016.1274657 28845097 PMC5568008

[63] Gu J Strauss C Bond R Cavanagh K. How do mindfulness-based cognitive therapy and mindfulness-based stress reduction improve mental health and wellbeing? A systematic review and meta-analysis of mediation studies. Clin Psychol Rev. (2015) 37:1–12. 10.1016/j.cpr.2015.01.006 25689576

[64] Ronk FR Hooke GR Page AC. How consistent are clinical significance classifications when calculation methods and outcome measures differ? Clin Psychol Sci Pract. (2012) 19:167–79. 10.1111/j.1468-2850.2012.01281.x

[65] Zeng X Oei TP Ye Y Liu X. A critical analysis of the concepts and measurement of awareness and equanimity in Goenka's Vipassana meditation. J Relig Health. (2015) 54:399–412. 10.1007/s10943-013-9796-9 24222100

[66] King AP Erickson TM Giardino ND Favorite T Rauch SA Robinson E. A pilot study of group mindfulness-based cognitive therapy (MBCT) for combat veterans with posttraumatic stress disorder (PTSD). Depress Anxiety. (2013) 30:638–45. 10.1002/da.22104 23596092 PMC4373594

[67] Piet J Hougaard E Hecksher MS Rosenberg NK. A randomized pilot study of mindfulness-based cognitive therapy and group cognitive-behavioral therapy for young adults with social phobia. Scand J Psychol. (2010) 51:403–10. 10.1111/j.1467-9450.2009.00801.x 20210911

[68] Crane C Crane RS Eames C Fennell MJV Silverton S Williams JMG. The effects of amount of home meditation practice in Mindfulness Based Cognitive Therapy on hazard of relapse to depression in the Staying Well after Depression Trial. Behav Res Ther. (2014) 63:17–24. 10.1016/j.brat.2014.08.015 25261599 PMC4271738

[69] Grabovac A. The stages of insight: clinical relevance for mindfulenss-based interventions. Mindfulness. (2014) 6:589–600. 10.1007/s12671-014-0294-2

[70] Harel O Hadash Y Levi-Belz Y Bernstein A. Does early emotional responding to initial mindfulness training impact intervention outcomes? Mindfulness. (2019) 10:616–26. 10.1007/s12671-018-1018-9

[71] Gárriz M Elices M Peretó M Martín-López LM Justicia A Pérez V. Mindfulness-based cognitive therapy delivered in primary care: a naturalistic, mixed-methods study of participant characteristics and experiences. Mindfulness. (2020) 11:291–302. 10.1007/s12671-019-01166-y

[72] Australian Bureau of Statistics. Australians Pursuing Higher Education in Record Numbers [Media Release]. ABS (2017). Available online at: https://www.abs.gov.au/Ausstats/abs@.nsf/dd0ca10eed681f12ca2570ce0082655d/1533fe5a8541d66cca2581bf00362d1d!OpenDocument (accessed May 12, 2020).

[73] Meadows GN Shawyer F Enticott JC Graham AL Judd F Martin PR. Mindfulness-based cognitive therapy for recurrent depression: a translational research study with 2-year follow-up. Austr N Zeal J Psychiatry. (2014) 48:743–55. 10.1177/0004867414525841 24595511

