# Supplementary material for: Group Mindfulness-Integrated Cognitive Behavior Therapy (MiCBT) Reduces Depression and Anxiety and Improves Flourishing in a Transdiagnostic Primary Care Sample Compared to Treatment-as-Usual: A Randomized Controlled Trial
Source: Front Psychiatry. 2022 May 31;13:815170. doi: 10.3389/fpsyt.2022.815170 (PMC9193586; doi:10.3389/fpsyt.2022.815170)
Supplement: Supplementary file 1 [file Data_Sheet_1.docx]

Supplementary Figure and Tables

# Supplementary Figures

Path ab_2_ = .17(.14), 95% CI [-.07, .48]

Path ab_1_ = .16(.15), 95% CI [-.13, .48]

Path a_1_= .89(.20)***

Path a_2_= .83(.19)***

Path b_2_ = .21(.17)

Path b_3_ = .13(.15)

Path a_3_= .73(.20)***

Path b_1_ = .18(.15)

MSES-R-E change (T2)

EQ change (T2)

FS change (T3)

Group

MSES-R-S change (T2)

Path ab_3_ = .10(.14), 95% CI [-.13, .41]

Path c’= -.23(.20)

Path c = .66 (.19)**

**Supplementary Figure 1.** Path diagram depicting model 2, testing whether changes in equanimity (MSES-R-E), awareness (EQ) and interpersonal skills (MSES-R-S) mediate the effects of MiCBT versus control on improvements in flourishing. Unstandardized path coefficients are displayed.

Notes. ** <.01; ***<.001; EQ = Experiences Questionnaire; MSES-R-E = Mindfulness-based Self-Efficacy Scale-Revised-Equanimity subscales; MSES-R-S = Mindfulness-based Self-Efficacy Scale-Revised-Interpersonal skills subscales; DASS-21 = Depression, Anxiety, and Stress Scale-21.

a, b, c’ and c are unstandardized regression coefficients (with standard errors) which represent predicting the mediator from group (a), DASS-21 change from M controlling for group (b), DASS-21 change from group controlling for the mediators (direct effect, c’), and DASS-21 change from group *not* controlling for the mediators (total effect, c). The product of a and b paths, ab, represents the mediated or indirect effect. Change refers to standardized residualized change scores.

# Supplementary Tables

**Supplementary Table 1.** Mean (SD, 95% CI) and median values of outcome and mediator variables for MiCBT and control groups from baseline (T0) to mid-treatment (T1), and of treatment (T2) and 6-month follow-up (t3)

| **Measure** |  | **T0** | | | **T1** | | | | **T2** | | | **T3** | | |
| --- | --- | --- | --- | --- | --- | --- | --- | --- | --- | --- | --- | --- | --- | --- |
|  |  | **Mean (SD)** | **Median** | **N** | **Mean (SD)** | **Median** | | **N** | **Mean (SD)** | **Median** | **N** | **Mean (SD)** | **Median** | **N** |
|  |  | (95% CI) |  |  | (95% CI) |  | |  | (95% CI) |  |  | (95% CI) |  |  |
| DASS-21 | MiCBT | 48.5 (23.1) | 46 | 60 | 35.4 (17.5) | 35 | | 50 | 30.1 (20.6) | 24 | 47 | 34.7 (22.8) | 30 | 49 |
|  |  | (42.5, 54.5) |  |  | (30.5, 40.4) |  |  |  | (24.7, 36.2) |  |  | (28.18, 41.3) |  |  |
|  |  |  |  |  |  |  | |  |  |  |  |  |  |  |
|  | Control | 50.2 (22.0) | 46 | 57 | 48.3 (27.5) | 46 | | 55 | 45.6 (23.6) | 44 | 47 | 44.7 (25.2) | 40 | 48 |
|  |  | (44.4, 56.0) |  |  | (48.3, 40.9) |  |  |  | (38.7, 52.6) |  |  | (37.3, 52.0) |  |  |
|  |  |  |  |  |  |  | |  |  |  |  |  |  |  |
| K10 | MiCBT | 27.8 (7.0) | 28 | 61 | 23.0 (6.8) | 21 | | 50 | 21.6 (7.9) | 20 | 45 | 21.6 (7.1) | 20 | 47 |
|  |  | (26.0, 29.5) |  |  | (21.1, 25.0) |  |  |  | (19.2, 24.0) |  |  | (19.5, 23.7) |  |  |
|  |  |  |  |  |  |  | |  |  |  |  |  |  |  |
|  | Control | 29.0 (6.3) | 30 | 57 | 26.8 (7.9) | 26 | | 53 | 27.2 (8.1) | 27 | 45 | 26.3 (8.1) | 26 | 47 |
|  |  | (27.3, 30.7) |  |  | (24.6, 29) |  |  |  | (24.8, 29.6) |  |  | (23.9, 28.6) |  |  |
|  |  |  |  |  |  |  | |  |  |  |  |  |  |  |
| SWLS | MiCBT | 17.1 (7.0) | 17 | 60 | 20.4 (6.6) | 22 | | 50 | 21.5 (6.7) | 22 | 47 | 21.8(6.5) | 22 | 48 |
|  |  | (15.3, 19.0) |  |  | (18.5, 22.2) |  |  |  | (19.5, 23.4) |  |  | (19.9, 23.7) |  |  |
|  |  |  |  |  |  |  | |  |  |  |  |  |  |  |
|  | Control | 17.7 (6.8) | 17 | 57 | 19.2 (7.8) | 19 | | 54 | 19.7 (7.4) | 20 | 47 | 18.9 (7.5) | 19 | 48 |
|  |  | (15.9, 19.5) |  |  | (17.1, 21.3) |  |  |  | (17.5, 21.8) |  |  | (16.7, 21.0) |  |  |
|  |  |  |  |  |  |  | |  |  |  |  |  |  |  |
| FS | MiCBT | 36.7 (9.2) | 36 | 60 | 40.4 (8.1) | 42 | | 50 | 42.4 (7.7) | 43 | 47 | 42.6 (7.0) | 43 | 48 |
|  |  | (33.3, 38.0) |  |  | (38.1, 42.7) |  |  |  | (40.2, 44.7) |  |  | (40.6, 44.7) |  |  |
|  |  |  |  |  |  |  | |  |  |  |  |  |  |  |
|  | Control | 36.1(8.6) | 36 | 57 | 37.7 (8.7) | 37 | | 54 | 37.2 (10.0) | 39 | 47 | 37.5 (9.6) | 40 | 48 |
|  |  | (33.8, 38.4) |  |  | (35.4, 40.1) |  |  |  | (34.3, 40.1) |  |  | (34.6, 40.2) |  |  |
|  |  |  |  |  |  |  | |  |  |  |  |  |  |  |
| MAIA | MiCBT | 65.2 (17.18) | 64 | 60 | 75.17 (14.9) | 82 | 50 | | 83.2 (18.2) | 87 | 45 | 77.1 (17.6) | 76 | 47 |
|  |  | 60.7, 69.6) |  |  | (75.2, 83.6) |  |  |  | (77.8, 88.7) |  |  | (71.9, 82.3) |  |  |
|  |  |  |  |  |  |  |  | |  |  |  |  |  |  |
|  | Control | 62.0 (15.1) | 64 | 57 | 61.59 (15.1) | 61 | 53 | | 61.7 (16.8) | 62 | 45 | 63.0 (17.1) | 62 | 47 |
|  |  | (58.0, 66.0) |  |  | (57.4, 65.7) |  |  |  | (56.7, 66.7) |  |  | (57.3, 67.3) |  |  |
|  |  |  |  |  |  |  |  | |  |  |  |  |  |  |
| NAS | MiCBT | 3.5 (.8) | 3.6 | 60 | 4.0 (.7) | 4.2 | 52 | | 4.3 (.8) | 4.5 | 45 | 4.3 (.7) | 4.3 | 47 |
|  |  | (3.3, 3.7) |  |  | (3.8, 4.2) |  |  |  | (4.0, 4.5) |  |  | (4.1, 4.5) |  |  |
|  |  |  |  |  |  |  |  | |  |  |  |  |  |  |
|  | Control | 3.6 (.79) | 3.6 | 57 | 3.5(.7) | 3.6 | 54 | | 3.6 (.7) | 3.6 | 45 | 3.7 (.8) | 3.7 | 47 |
|  |  | (3.4, 3.8) |  |  | (3.4, 3.7) |  |  |  | (3.3, 3.8) |  |  | (3.5, 4.0) |  |  |
|  |  |  |  |  |  |  |  | |  |  |  |  |  |  |
| EQ | MiCBT | 30.6 (5.8) | 31 | 60 | 34.6 (5.0) | 35 | 50 | | 36.0 (6.4) | 37 | 47 | 36.4 (5.8) | 37 | 48 |
|  |  | (29.1, 32.0) |  |  | (33.2, 36.0) |  |  |  | (34.2, 37.9) |  |  | (34.7, 38.1) |  |  |
|  |  |  |  |  |  |  |  | |  |  |  |  |  |  |
|  | Control | 29.2 (5 .3) | 28 | 57 | 29.7 (5.8) | 30 | 53 | | 30.0 (6.0) | 29 | 46 | 30.92 (6.7) | 31 | 48 |
|  |  | (27.8, 30.6) |  |  | (28.1, 31.3) |  |  |  | (28.3, 31.8) |  |  | (29.0, 32.8) |  |  |
| MSES - total | MiCBT | 47.0 (9.02) (44.6, 49.3) | 45 | 60 | 54.8 (9.2) (52.2, 57.5) | 55 | 50 | | 58.8 (11.2) (55.5, 62.1) | 60 | 47 | 59.2 (11.0) (56.0, 62.3) | 60 | 48 |
|  | Control | 47.40(10.7)(44.6, 50.2) | 49 | 57 | 48 (11.0) (45.0, 51.0) | 49 | 55 | | 49.1 (11.4) (45.6, 52.5) | 49 | 47 | 49.2 (11.7) (45.8, 52.6) | 49 | 48 |
| MSES Equanimity | MiCBT | 25.6 (6.2) (24.0, 27.2) | 25 | 60 | 30.3 (6.7) (28.4, 32.2) | 31 | 50 | | 32.6 (7.6) (30.6, 35.0) | 32 | 47 | 33.3 (7.4) 31.1, 35.4) | 34 | 48 |
|  | Control | 25.6 (7.0) (23.8, 27.5) | 26 | 57 | 25.7 (7.2) (23.7, 27.6) | 26 | 55 | | 26.4 (7.2) (24.3, 28.5) | 26 | 47 | 26.8 (6.89 (24.1, 28.8) | 27 | 48 |
| MSES Interpersonal | MiCBT | 20.2 (4.5) (20.2, 22.5) | 22 | 60 | 24.6 (3.9) (23.5, 25.7) | 26 | 50 | | 26.0 (4.5) (24.7, 27.3) | 27 | 47 | 25.5 (4.5) (24.2, 26.8) | 26 | 48 |
|  | Control | 21.78(5.4) (20.4, 23.2) | 21 | 57 | 22.4 (5.4) (21.0, 23.8) | 24 | 55 | | 22.7 (5.7) (21.1, 24.4) | 23 | 47 | 21.9 (5.8) (20.2, 23.6) | 22 | 48 |

Note. DASS-21- Depression, Anxiety and Stress Scale; K10 = Kessler Psychological Distress Scale; SWLS = Satisfaction with Life Scale; FS = Flourishing Scale; MAIA - Multidimensional Assessment of Interoceptive Awareness; NAS - Non-attachment Scale; EQ - Experiences Questionnaire; MSES-E - Mindfulness-based Self-efficacy Scale.

**Supplementary Table 2**. Mean (SD, 95%CI) and median values of DASS-21 Subscales (Depression, Anxiety and Stress) for MiCBT and control groups from baseline (T0) to mid-treatment (T1), and of treatment (T2) and 6-month follow-up (T3)

| **Measure** |  | **T0** | | | **T1** | | | **T2** | | | **T3** | | |
| --- | --- | --- | --- | --- | --- | --- | --- | --- | --- | --- | --- | --- | --- |
|  |  | **Mean (SD)** | **Median** | **N** | **Mean (SD)** | **Median** | **N** | **Mean (SD)** | **Median** | **N** | **Mean (SD)** | **Median** | **N** |
|  |  | (95% CI) |  |  | (95% CI) |  |  | (95% CI) |  |  | (95% CI) |  |  |
| DASS-21 | MiCBT | ^16.2 (10.93)^ | 15 | 60 | 11.6 (19.16) | 10 | 50 | 9.87 (9.26) | 6 | 47 | 12.12 (9.38) | 10 | 48 |
| Depression |  | ^(13.38, 19.02)^ |  |  | (9.0, 14.2) |  |  | (7.15, 12.59) |  |  | (9.43, 14.82) |  |  |
|  |  |  |  |  |  |  |  |  |  |  |  |  |  |
|  | Control | 17.02 (10.23) | 16 | 57 | 16.18 (11.86) | 14 | 55 | 15.32 (10.05) | 14 | 47 | 14.54 (10.32) | 13 | 49 |
|  |  | (14.3, 19.73) |  |  | (12.98, 19.39) |  |  | (12.37, 18.27) |  |  | (11.55, 17.54) |  |  |
|  |  |  |  |  |  |  |  |  |  |  |  |  |  |
| DASS-21 | MiCBT | 11.57 (7.68) | 10 | 60 | 8.36 (6.08) | 7 | 50 | 6.85 (6.1) | 6 | 47 | 8.20 (8.65) | 6 | 49 |
| Anxiety |  | (9.58, 13.55) |  |  | (6.33, 10.09) |  |  | (5.06, 8.64) |  |  | (5.72, 10.69) |  |  |
|  |  |  |  |  |  |  |  |  |  |  |  |  |  |
|  | Control | 11.6 (8.33) | 10 | 57 | 11.53 (9.59) | 10 | 55 | 10.26 (8.19) | 8 | 47 | 10.21 (9.35) | 8 | 48 |
|  |  | (9.4, 13.82) |  |  | (8.93, 14.12) |  |  | (7.85, 12.66) |  |  | (7.5, 12.92) |  |  |
| DASS-21 |  |  |  |  |  |  |  |  |  |  |  |  |  |
| Stress | MiCBT | 20.73 (8.8) | 21 | 60 | 15.48 (6.11) | 16 | 50 | 13.4 (7.75) | 12 | 47 | 14.14 (8.38) | 14 | 48 |
|  |  | (18.46, 23.01) |  |  | (13.74, 17.22) |  |  | (11.13, 15.68) |  |  | (12.0, 16.81) |  |  |
|  |  |  |  |  |  |  |  |  |  |  |  |  |  |
|  | Control | 21.58 (8.34) | 22 | 57 | 20.62 (10.62) | 18 | 55 | 20.04 (8.96) | 20 | 47 | 19.92 (8.87) | 18 | 49 |
|  |  | (19.37, 23.79) |  |  | (17.75, 23.49) |  |  | (17.41, 22.67) |  |  | (17.34, 22.49) |  |  |

Note. DASS-21- Depression, Anxiety and Stress Scale

**Supplementary Table 3.** Mixed REML regression models for mediator variables with fixed factors of time and intervention status, and participants as random

| **MAIA** | **Timepoint** | **b** | **se** | **z** | **p value** | **[95%Conf.** | **Interval]** |
| --- | --- | --- | --- | --- | --- | --- | --- |
| Timepoint (compared to baseline)  T1 | | 6.67 | 1.52 | 4.38 | 0.000 | 3.69 | 9.65 |
| T2 | | 9.14 | 1.86 | 4.92 | 0.000 | 5.50 | 12.78 |
| T3 | | 6.06 | 1.81 | 3.36 | 0.001 | 2.52 | 9.60 |
| Intervention status | |  |  |  |  |  |  |
| MiCBT compared to control | | 12.23 | 1.10 | 11.17 | 0.000 | 10.09 | 14.38 |

*Number of observations =404, Number of participants = 118, Bootstrap replications = 50*

| **NAS** | **Timepoint** | b | **se** | **Z** | **p value** | **[95%Conf.** | **Interval]** |
| --- | --- | --- | --- | --- | --- | --- | --- |
| Timepoint (compared to baseline)  T1 | | 6.93 | 2.18 | 3.18 | 0.001 | 2.66 | 11.20 |
| T2 | | 12.28 | 2.05 | 5.98 | 0.000 | 8.25 | 16.30 |
| T3 | | 13.65 | 2.24 | 6.09 | 0.000 | 9.26 | 18.05 |
| Intervention status | |  |  |  |  |  |  |
| MiCBT compared to control | | 11.47 | 1.40 | 8.21 | 0.000 | 8.73 | 14.21 |

*Number of observations =404, Number of participants = 118, Bootstrap replications = 50*

| **EQ** | **Timepoint** | **b** | **se** | **z** | **p value** | **[95%Conf.** | **Interval]** |
| --- | --- | --- | --- | --- | --- | --- | --- |
| Timepoint (compared to baseline)  T1 | | 2.18 | .51 | 4.29 | 0.000 | 1.18 | 3.18 |
| T2 | | 3.40 | .61 | 5.54 | 0.000 | 2.19 | 4.60 |
| T3 | | 3.83 | .58 | 6.65 | 0.000 | 2.70 | 4.96 |
| Intervention status | |  |  |  |  |  |  |
| MiCBT compared to control | | 3.83 | .45 | 8.55 | 0.000 | 2.95 | 4.71 |

*Number of observations =409, Number of participants = 118, Bootstrap replications = 50*

| **MSES R- E** **Timepoint** |  | b | se | Z | p value | [95%Conf. | Interval] |
| --- | --- | --- | --- | --- | --- | --- | --- |
| Timepoint (compared to baseline)  T1 | | 2.36 | .56 | 4.24 | 0.000 | 1.27 | 3.45 |
| T2 | | 4.24 | .63 | 6.76 | 0.000 | 3.01 | 5.47 |
| T3 | | 4.81 | .68 | 7.08 | 0.000 | 3.48 | 6.14 |
| Intervention status | |  |  |  |  |  |  |
| MiCBT compared to control | | 3.80 | .56 | 6.83 | 0.000 | 2.71 | 4.88 |

*Number of observations =412, Number of participants = 118, Bootstrap replications = 50*

| **MSES R-S** | **Timepoint** | b | se | z | p value | [95%Conf. | Interval] |
| --- | --- | --- | --- | --- | --- | --- | --- |
| Timepoint (compared to baseline)  T1 | | 1.79 | .36 | 4.99 | 0.000 | 1.09 | 2.49 |
| T2 | | 2.90 | .44 | 6.58 | 0.000 | 2.04 | 3.77 |
| T3 | | 2.39 | .49 | 4.84 | 0.000 | 1.42 | 3.35 |
| Intervention status | |  |  |  |  |  |  |
| MiCBT compared to control | | 1.79 | .37 | 4.88 | 0.000 | 1.07 | 2.51 |

*Number of observations =412, Number of participants = 118, Bootstrap replications = 50*

**Supplementary Table 4.** Mixed effect regression using Multiple Imputation with Chained Equations (MICE)

| **DASS-21** | **b** | **se** | **t** | **p value** | **[95%Conf.** | **Interval]** |
| --- | --- | --- | --- | --- | --- | --- |
| Timepoint |  |  |  |  |  |  |
|  |  |  |  |  |  |  |
| T1 | -6.77 | 2.23 | -3.04 | 0.002 | -11.14 | -2.41 |
| T2 | -10.26 | 2.46 | -4.16 | 0.000 | -15.12 | -5.39 |
| T3 | -9.20 | 2.32 | -3.97 | 0.000 | -13.75 | -4.65 |
| Intervention status |  |  |  |  |  |  |
| 1 | -8.20 | 3.36 | -2.44 | 0.015 | -14.80 | -1.61 |

Notes. Number of imputations = 10, Number of observations = 472 (complete = 413, incomplete = 59, imputed = 59), Number of participants = 118, Observations per participant = 4, DF min = 40.16; Average = 45,810.37, Maxx = 296,057.19, Prob > F = 0.0000.

**Supplementary Table 5*.*** Correlation between Mediation Model Variables at Baseline (sample size in parentheses)

|  | **NAS T0** | **MSES-E T0** | **FS T0** | **MAIA T0** | **MSES-S T0** | **DASS-21 T0** | **FS T0** |
| --- | --- | --- | --- | --- | --- | --- | --- |
| Group | __ |  |  |  |  |  |  |
| NAS T0 | 1.00 (117) | __ |  |  |  |  |  |
| MSES-E T0 | .35** (117) | 1.00 (117) | __ |  |  |  |  |
| EQ T0 | .55** (117) | .30** (117) | 1.00 (117) | __ |  |  |  |
| MAIA T0 | .49** (117) | .30** (117) | .72** (117) | 1.00 (117) | __ |  |  |
| MSES-S T0 | .40** (117) | .24* (117) | .22* (117) | .25** (117) | 1.00 (117) | __ |  |
| DASS-21 T0 | -.35** (117) | -.09 (117) | -.28** (117) | .28 (117) | -.36** (117) | 1.00 (117) | __ |
| FS T0 | .46** (117) | .26** (117) | .44** (117) | .38** (117) | .53** (117) | -.44** (117) | 1.00 (117) |

Note. Pearson’s r correlation coefficients, with sample size (n) in parentheses.

**p* < 0.05; ** *p* < 0.01

**Supplementary Table 6*.*** Correlation between Mediation Model Variables (sample size in parentheses)

|  | **NAS T2** | **MSES-E T2** | **EQ**  **T2** | **MAIA T2** | **MSES-S T2** | **DASS-21 T3** | **FS**  **T3** |
| --- | --- | --- | --- | --- | --- | --- | --- |
| NAS T2 | 1.00 (90) | — |  |  |  |  |  |
| MSES-E T2 | .75*** (90) | 1.00 (94) | — |  |  |  |  |
| EQ T2 | .73*** (90) | .78*** (93) | 1.00 (93)) | — |  |  |  |
| MAIA T2 | .67*** (90) | .67*** (90) | .85*** (90) | 1.00 (90) | — |  |  |
| MSES-S T2 | .65*** (90) | .71*** (94) | .56*** (93) | .45*** (90) | 1.00 (94) | — |  |
| DASS-21 T3 | -.39*** (87) | -.54*** (89) | -.41*** (89) | -.34** (87) | -.57*** (89) | 1.00 (97) | — |
| FS T3 | .45*** (87) | .46*** (89) | .46*** (89) | .40*** (87) | .62*** (89) | -.58*** (96) | 1.00 (96) |

Note. Pearson’s r correlation coefficients, with sample size (n) in parentheses.

** *p* < 0.01; *** *p* < 0.00

**Supplementary Table 7.** Unstandardized regression coefficients, their standard errors (SEs) and significance values, and bootstrapped unstandardized point estimates and their SEs and 95% confidence intervals, for the four independent variable mediation models assessing change in the primary outcome (DASS-21) during the follow-up period (mediators measured at 8 weeks; outcomes at 6 months)

| **Model** | **B** | **SE** | **t** | **p** | **Indirect effect point estimate (SE) [95% CIs]^a^** | **Percentage of total effect that operates indirectly** |
| --- | --- | --- | --- | --- | --- | --- |
| Model 1: with MAIA as the mediator (*n* = 86) |  |  |  |  | -9.54 (3.07) [-16.26, -4.18] | 85.8% |
| *a_1_* path: Group - MAIA | 19.17 | 3.31 | 5.80 | .00 |  |  |
| *b_1_* path: MAIA - DASS-21 | -.50 | .14 | -3.55 | .00 |  |  |
| *c_1_* path: Group - DASS-21(total effect) | -11.12 | 4.49 | -2.48 | .02 |  |  |
| *c'_1_* path: Group - DASS-21(direct effect) | -1.58 | 4.99 | -.32 | .75 |  |  |
| Model 2: with EQ as the mediator (*n* = 88) |  |  |  |  | -6.87 (3.41) [-14.29, -1.04] | 67.6% |
| *a_1_* path: Group - EQ | 5.28 | 1.15 | 4.59 | .00 |  |  |
| *b_1_* path: EQ - DASS-21 | -1.30 | .40 | -3.24 | .00 |  |  |
| *c_1_* path: Group - DASS-21(total effect) | -10.17 | 4.46 | -2.28 | .03 |  |  |
| *c'_1_* path: Group - DASS-21(direct effect) | -3.30 | 4.73 | -.70 | .49 |  |  |
| Model 3: with NAS as the mediator (*n* = 86) |  |  |  |  | -10.05 (2.99) [-16.46, -4.59] | 93.3% |
| *a_1_* path: Group - NAS | 21.02 | 3.60 | 5.84 | .00 |  |  |
| *b_1_* path: NAS - DASS-21 | -.48 | .13 | -3.79 | .00 |  |  |
| *c_1_* path: Group - DASS-21(total effect) | -10.77 | 4.43 | -2.43 | .02 |  |  |
| *c'_1_* path: Group - DASS-21(direct effect) | -.71 | 4.89 | 0.15 | .88 |  |  |
| Model 4: with MSES-E as the mediator (*n* = 88) |  |  |  |  | -7.90 (3.34) [-15.37, -2.41) | 75.2% |
| *a_1_* path: Group - MSES-E | 5.93 | 1.38 | 4.30 | .00 |  |  |
| *b_1_* path: MSES-E - DASS-21 | -1.33 | .32 | -4.15 | .00 |  |  |
| *c_1_* path: Group - DASS-21(total effect) | -10.50 | 4.42 | -2.37 | .02 |  |  |
| *c'_1_* path: Group - DASS-21(direct effect) | -2.60 | 4.47 | -.58 | .56 |  |  |

^a^Bootstrapped 95% BC CIs for the ab (indirect) effect; a significant indirect effect is indicated where these do not cross zero (p < .05).

Note. DASS-21- Depression, Anxiety and Stress Scale; MAIS - Multidimensional Assessment of Interoceptive Awareness; EQ - Experiences Questionnaire; NAS - Non-attachment Scale; MSES-E - Mindfulness-based Self-efficacy Scale (Equanimity subscales).

**Supplementary Table 8.** Unstandardized regression coefficients, their standard errors (SEs) and significance values, and bootstrapped unstandardized point estimates and their SEs and 95% confidence intervals, for the five independent variable mediation models assessing change in the Flourishing Scale during the follow-up period (mediators measured at 8 weeks; outcomes at 6 months)

| **Model** | **B** | **SE** | **t** | **p** | **Indirect effect point estimate (SE) [95% CIs]^a^** | **Percentage of total effect that operates indirectly** |
| --- | --- | --- | --- | --- | --- | --- |
| Model 1: with MAIA as the mediator (n = 86) |  |  |  |  | 2.69 (1.00) [.82, 7.72] | 57.2% |
| *a_1_* path: Group - MAIA | 19.10 | 3.29 | 5.80 | .00 |  |  |
| *b_1_* path: MAIA - FS | .14 | .04 | 3.30 | .00 |  |  |
| *c_1_* path: Group - FS (total effect) | 4.70 | 1.35 | 3.48 | .00 |  |  |
| *c'_1_* path: Group - FS (direct effect) | 2.01 | 1.52 | 1.32 | .19 |  |  |
| Model 2: with EQ as the mediator (*n* = 88) |  |  |  |  | 2.57 (.89) [1.02, 4.51] | 57.1% |
| *a_1_* path: Group - EQ | 5.27 | 1.16 | 4.56 | .00 |  |  |
| *b_1_* path: EQ - FS | .49 | .11 | 4.27 | .00 |  |  |
| *c_1_* path: Group - FS (total effect) | 4.50 | 1.33 | 3.38 | .00 |  |  |
| *c'_1_* path: Group - FS (direct effect) | 1.93 | 1.35 | 1.42 | .16 |  |  |
| Model 3: with NAS as the mediator (*n* = 86) |  |  |  |  | 3.68 (.95) [1.94, 5.61] | 79.3% |
| *a_1_* path: Group - NAS | 21.20 | 3.60 | 5.90 | .00 |  |  |
| *b_1_* path: NAS - FS | .17 | .04 | 4.75 | .00 |  |  |
| *c_1_* path: Group - FS (total effect) | 4.64 | 1.34 | 3.47 | .00 |  |  |
| *c'_1_* path: Group - FS (direct effect) | .96 | 1.42 | .68 | .50 |  |  |
| Model 4: with MSES-E as the mediator (*n* = 88) |  |  |  |  | 2.51 (.91) [1.02, 4.51] | 56.8% |
| *a_1_* path: Group - MSES-E | 5.93 | 1.39 | 4.26 | .00 |  |  |
| *b_1_* path: MSES-E - FS | .42 | .09 | 4.56 | .00 |  |  |
| *c_1_* path: Group - FS (total effect) | 4.42 | 1.31 | 3.37 | .00 |  |  |
| *c'_1_* path: Group - FS (direct effect) | 1.92 | 1.30 | 1.47 | .14 |  |  |
| Model 5: with MSES-S as the mediator (*n* = 88) |  |  |  |  | 2.05 (.90) [.58, 4.02] | 54.4% |
| *a_1_* path: Group - MSES-S | 3.13 | .88 | 3.56 | .00 |  |  |
| *b_1_* path: MSES-S - FS | .66 | .14 | 4.53 | .00 |  |  |
| *c_1_* path: Group - FS (total effect) | 4.50 | 1.29 | 3.49 | .00 |  |  |
| *c'_1_* path: Group - FS (direct effect) | 2.45 | 1.25 | 1.97 | .05 |  |  |

^a^Bootstrapped 95% BC CIs for the ab (indirect) effect; a significant indirect effect is indicated where these do not cross zero (p < .05).

Note. FS - Flourishing Scale; MAIS - Multidimensional Assessment of Interoceptive Awareness; EQ - Experiences Questionnaire; NAS - Non-attachment Scale; MSES-E - Mindfulness-based Self-efficacy Scale (Equanimity subscales); MSES-S - Mindfulness-based Self-efficacy Scale (Social skills subscales).

**Supplementary Table 9.** SPSS output from parallel mediation model 1 testing whether changes in equanimity (MSESe) and metacognitive awareness (EQ) mediate the effects of MiCBT versus control on improvements in the DASS-21.

Run MATRIX procedure:

***************** PROCESS Procedure for SPSS Version 3.5 *****************

Written by Andrew F. Hayes, Ph.D. www.afhayes.com

Documentation available in Hayes (2018). www.guilford.com/p/hayes3

**************************************************************************

Model : 4

Y : ZDASt4_1

X : GROUP

M1 : ZEQt3_1

M2 : ZMSEe3_1

Sample

Size: 88

**************************************************************************

OUTCOME VARIABLE:

ZEQt3_1

Model Summary

R R-sq MSE F df1 df2 p

.443 .196 .836 20.960 1.000 86.000 .000

Model

coeff se t p LLCI ULCI

constant -.436 .138 -3.164 .002 -.710 -.162

GROUP .892 .195 4.578 .000 .505 1.280

**************************************************************************

OUTCOME VARIABLE:

ZMSEe3_1

Model Summary

R R-sq MSE F df1 df2 p

.420 .177 .828 18.471 1.000 86.000 .000

Model

coeff se t p LLCI ULCI

constant -.428 .137 -3.119 .002 -.701 -.155

GROUP .834 .194 4.298 .000 .448 1.220

**************************************************************************

OUTCOME VARIABLE:

ZDASt4_1

Model Summary

R R-sq MSE F df1 df2 p

.469 .220 .802 7.887 3.000 84.000 .000

Model

coeff se t p LLCI ULCI

constant .039 .144 .272 .786 -.247 .325

GROUP -.120 .215 -.560 .577 -.547 .307

ZEQt3_1 -.061 .159 -.382 .703 -.376 .255

ZMSEe3_1 -.388 .159 -2.437 .017 -.705 -.071

************************** TOTAL EFFECT MODEL ****************************

OUTCOME VARIABLE:

ZDASt4_1

Model Summary

R R-sq MSE F df1 df2 p

.251 .063 .941 5.802 1.000 86.000 .018

Model

coeff se t p LLCI ULCI

constant .232 .146 1.584 .117 -.059 .522

GROUP -.498 .207 -2.409 .018 -.909 -.087

************** TOTAL, DIRECT, AND INDIRECT EFFECTS OF X ON Y **************

Total effect of X on Y

Effect se t p LLCI ULCI c_ps

-.498 .207 -2.409 .018 -.909 -.087 -.500

Direct effect of X on Y

Effect se t p LLCI ULCI c'_ps

-.120 .215 -.560 .577 -.547 .307 -.121

Indirect effect(s) of X on Y:

Effect BootSE BootLLCI BootULCI

TOTAL -.378 .174 -.755 -.079

ZEQt3_1 -.054 .144 -.348 .233

ZMSEe3_1 -.324 .157 -.679 -.067

Partially standardized indirect effect(s) of X on Y:

Effect BootSE BootLLCI BootULCI

TOTAL -.379 .176 -.756 -.081

ZEQt3_1 -.054 .147 -.357 .233

ZMSEe3_1 -.325 .155 -.672 -.069

*********************** ANALYSIS NOTES AND ERRORS ************************

Level of confidence for all confidence intervals in output:

95.0000

Number of bootstrap samples for percentile bootstrap confidence intervals:

5000

------ END MATRIX -----

Note: 4_1 = T3 and 3_1 = T2 in this analysis.

**Supplementary Table 10.** SPSS output from parallel mediation model 2 testing whether changes in interpersonal skills (MSESs) equanimity (MSESe) and metacognitive awareness (EQ) mediate the effects of MiCBT versus control on improvements in the DASS-21.

Run MATRIX procedure:

***************** PROCESS Procedure for SPSS Version 3.5 *****************

Written by Andrew F. Hayes, Ph.D. www.afhayes.com

Documentation available in Hayes (2018). www.guilford.com/p/hayes3

**************************************************************************

Model : 4

Y : ZFS4_1

X : GROUP

M1 : ZEQt3_1

M2 : ZMSEe3_1

M3 : ZMSEs3_1

Sample

Size: 88

**************************************************************************

OUTCOME VARIABLE:

ZEQt3_1

Model Summary

R R-sq MSE F df1 df2 p

.443 .196 .836 20.960 1.000 86.000 .000

Model

coeff se t p LLCI ULCI

constant -.436 .138 -3.164 .002 -.710 -.162

GROUP .892 .195 4.578 .000 .505 1.280

**************************************************************************

OUTCOME VARIABLE:

ZMSEe3_1

Model Summary

R R-sq MSE F df1 df2 p

.420 .177 .828 18.471 1.000 86.000 .000

Model

coeff se t p LLCI ULCI

constant -.428 .137 -3.119 .002 -.701 -.155

GROUP .834 .194 4.298 .000 .448 1.220

**************************************************************************

OUTCOME VARIABLE:

ZMSEs3_1

Model Summary

R R-sq MSE F df1 df2 p

.365 .133 .877 13.214 1.000 86.000 .000

Model

coeff se t p LLCI ULCI

constant -.354 .141 -2.505 .014 -.634 -.073

GROUP .726 .200 3.635 .000 .329 1.123

**************************************************************************

OUTCOME VARIABLE:

ZFS4_1

Model Summary

R R-sq MSE F df1 df2 p

.562 .316 .668 9.585 4.000 83.000 .000

Model

coeff se t p LLCI ULCI

constant -.098 .131 -.749 .456 -.359 .163

GROUP .233 .196 1.189 .238 -.157 .623

ZEQt3_1 .179 .150 1.192 .237 -.120 .478

ZMSEe3_1 .209 .168 1.245 .217 -.125 .542

ZMSEs3_1 .131 .147 .887 .378 -.162 .423

************************** TOTAL EFFECT MODEL ****************************

OUTCOME VARIABLE:

ZFS4_1

Model Summary

R R-sq MSE F df1 df2 p

.345 .119 .831 11.587 1.000 86.000 .001

Model

coeff se t p LLCI ULCI

constant -.312 .137 -2.269 .026 -.585 -.039

GROUP .662 .194 3.404 .001 .275 1.048

************** TOTAL, DIRECT, AND INDIRECT EFFECTS OF X ON Y **************

Total effect of X on Y

Effect se t p LLCI ULCI c_ps

.662 .194 3.404 .001 .275 1.048 .685

Direct effect of X on Y

Effect se t p LLCI ULCI c'_ps

.233 .196 1.189 .238 -.157 .623 .241

Indirect effect(s) of X on Y:

Effect BootSE BootLLCI BootULCI

TOTAL .429 .149 .188 .766

ZEQt3_1 .160 .151 -.130 .475

ZMSEe3_1 .174 .142 -.073 .480

ZMSEs3_1 .095 .136 -.128 .411

(C1) -.014 .243 -.507 .466

(C2) .065 .228 -.422 .489

(C3) .079 .228 -.389 .526

Partially standardized indirect effect(s) of X on Y:

Effect BootSE BootLLCI BootULCI

TOTAL .444 .132 .215 .728

ZEQt3_1 .165 .154 -.147 .473

ZMSEe3_1 .180 .145 -.080 .487

ZMSEs3_1 .098 .140 -.134 .420

(C1) -.015 .253 -.534 .479

(C2) .067 .237 -.454 .486

(C3) .082 .235 -.400 .544

Specific indirect effect contrast definition(s):

(C1) ZEQt3_1 minus ZMSEe3_1

(C2) ZEQt3_1 minus ZMSEs3_1

(C3) ZMSEe3_1 minus ZMSEs3_1

*********************** ANALYSIS NOTES AND ERRORS ************************

Level of confidence for all confidence intervals in output:

95.0000

Number of bootstrap samples for percentile bootstrap confidence intervals:

5000

------ END MATRIX -----

Note: 4_1 = T3 and 3_1 = T2 in this analysis.
